# Comparison of Three Viral Nucleic Acid Preamplification Pipelines for Sewage Viral Metagenomics

**DOI:** 10.1007/s12560-024-09594-3

**Published:** 2024-04-22

**Authors:** Xavier Fernandez-Cassi, Tamar Kohn

**Affiliations:** 1 Laboratory of Environmental Chemistry, School of Architecture, Civil and Environmental Engineering, École Polytechnique Fédérale de Lausanne (EPFL), Vaud, Lausanne, Switzerland; 2 Departament of Biology, Healthcare and Environment, Faculty of Pharmacy and Food Sciences, University of Barcelona (UB), Barcelona, Catalunya Spain

**Keywords:** Viral metagenomics, Sewage monitoring, Environmental surveillance, Enteric viruses, Waterborne pathogens

## Abstract

Viral metagenomics is a useful tool for detecting multiple human viruses in urban sewage. However, more refined protocols are required for its effective use in disease surveillance. In this study, we investigated the performance of three different preamplification pipelines (specific to RNA viruses, DNA viruses or both) for viral genome sequencing using spiked-in Phosphate Buffered Saline and sewage samples containing known concentrations of viruses. We found that compared to the pipeline targeting all genome types, the RNA pipeline performed better in detecting RNA viruses in both spiked and unspiked sewage samples, allowing the detection of various mammalian viruses including members from the *Reoviridae*, *Picornaviridae*, *Astroviridae* and *Caliciviridae*. However, the DNA-specific pipeline did not improve the detection of mammalian DNA viruses. We also measured viral recovery by quantitative reverse transcription polymerase chain reaction and assessed the impact of genetic background (non-viral genetic material) on viral coverage. Our results indicate that viral recoveries were generally lower in sewage (average of 11.0%) and higher in Phosphate Buffered Saline (average of 23.4%) for most viruses. Additionally, spiked-in viruses showed lower genome coverage in sewage, demonstrating the negative effect of genetic background on sequencing. Finally, correlation analysis revealed a relationship between virus concentration and genome normalized reads per million, indicating that viral metagenomic sequencing can be semiquantitative.

**Graphical Abstract:**

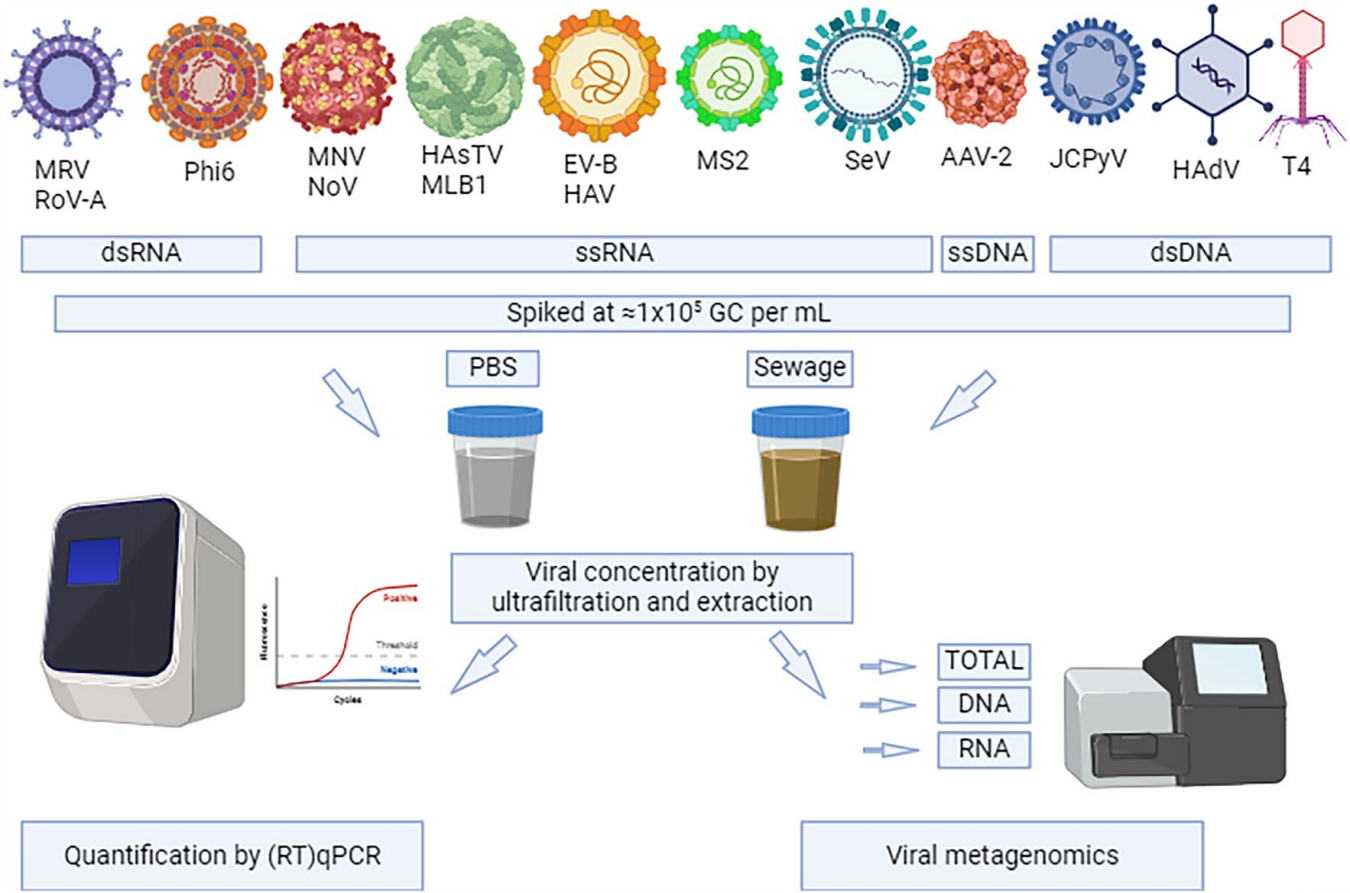

**Supplementary Information:**

The online version contains supplementary material available at 10.1007/s12560-024-09594-3.

## Introduction

Sewage contains a broad variety of viruses (Bibby & Peccia, 2013; Cantalupo et al., 2011; Ng et al., 2012) and has been suggested as a matrix for the surveillance of viruses circulating among a population (Fernandez-Cassi et al., 2018). Until recently, most of the commonly studied human viruses in sewage are enteric viruses with a fecal–oral transmission route such as noroviruses, enteroviruses or rotaviruses (Hellmér et al., 2014; Santiso-Bellón et al., 2020). Since the onset of the COVID-19 pandemic, however, sewage surveillance has also proven to be a useful tool to track the dynamics of respiratory viruses such as SARS-CoV-2 (Fernandez-Cassi et al., 2021; Randazzo et al., 2020), Respiratory Syncytial Virus (RSV) (Hughes et al., 2022), influenza (Heijnen & Medema, 2011) or monkeypox virus (de Jonge et al., 2022; Wolfe et al., 2023). Sewage has proven to be a useful tool to track the occurrence of variants (Jahn et al., 2021) and to derive epidemiological parameters of interest such as the effective reproductive number for SARS-CoV-2 as (Huisman et al., 2021). Accordingly, the interest in using sewage as a tool to monitor viral diseases has dramatically increased, and surely the use of this matrix will be further expanded in the near future to monitor other viral diseases previously unexplored.

Traditionally, viruses in sewage have been studied using quantitative PCR (qPCR) for specific viral pathogens or viral indicators (Hellmér et al., 2014; Kitajima et al., 2014; Santiso-Bellón et al., 2020). In the last decade due to the democratization of next-generation sequencing, virus surveillance has been done using amplicon sequencing methods to study the diversity within a specific viral family (Bisseux et al., 2020; Fernandez-Cassi et al., 2018; Iaconelli et al., 2017), as well as using non-targeted metaviromic studies to explore the viral diversity without aiming at any particular viruses (Cantalupo et al., 2011; Fernandez-Cassi et al., 2018; McCall et al., 2020; Ng et al., 2012). Both methodologies provide qualitative information (presence/absence) on pathogens but can also infer important genomic information pertaining to viral species or variants. However, amplicon sequencing approaches have the limitation that they are primer-dependent and hence can only be applied to known and expected viral targets. In contrast, metaviromic approaches are primer-independent, and can thus be used to detect a broader range of viruses, including new or unexpected species.

Despite its potential as a catch-all approach, there are several challenges to the application of sewage viral metagenomics as a routine method for viral surveillance. First, mammalian viruses are present in sewage at concentrations that are too low for direct sequencing. The sewage sample must first be concentrated and extracted, and nucleic acids must be preamplified in order to meet the requirements for library preparation. Both procedures can be a source of bias (Nieuwenhuijse et al., 2020; Parras-Moltó et al., 2018). Second, mammalian viruses in sewage are vastly outnumbered by both phages and phytoviruses (Bačnik et al., 2020; Cantalupo et al., 2011; Duarte et al., 2023), which are enriched along with mammalian viruses during sample concentration. In addition, sewage also contains a high background of nucleic acids from non-viral organisms. Nucleic acids from phages, phytoviruses and non-viral organisms are co-amplified and sequenced with the viral targets, thereby reducing the sequencing depth and the coverage of the viral species of interest (Krishnamurthy & Wang, 2017; Tamaki et al., 2012). Third, mammalian viruses present in sewage can contain different genome conformations (single- and double-stranded, linear and circular genomes), and their genomes can be codified using DNA or RNA molecules. This implies the need of molecular biology methodologies that work efficiently with both DNA and RNA molecules. And finally, methods used to concentrate viral particles from sewage tend to co-concentrate inhibitory substances that interfere with subsequent molecular biology reactions (Fernandez-Cassi et al., 2018; Schrader et al., 2012).

For viral metagenomics to serve as a tool to study the presence of multiple human viruses in a single analysis, refined protocols are thus needed to increase both the method sensitivity and its ability to specifically capture viruses. Several studies have been developing strategies to increase this sensitivity by applying specific viral probes to capture mammalian viruses after library preparation (Martínez-Puchol et al., 2020; Tisza et al., 2023). These approximations aim to enhance the library preparation for vertebrate viruses by excluding the sequencing of bacteriophages and other sources of DNA, thereby increasing the number of reads and the coverage of the targeted viruses of interest.

In recent years, significant progress has been made in developing systematic protocols and techniques for implementing viral metagenomics in a clinical setting (Conceição-Neto et al., 2018; Hall et al., 2014; Kohl et al., 2015; Li et al., 2015; Tulloch et al., 2023). Although there has been a growing interest in using sewage as a sample to monitor circulating viruses within a population, especially since the emergence of COVID-19, there is still a lack of specific protocols to assess viral diversity through viral metagenomics in this specific context.

In this study, we assess how viral metagenomic sequencing is affected by virus recovery, background genetic material, and preamplification protocols. We conducted metagenomic analyses in two scenarios: a low genetic background scenario using phosphate buffered saline (PBS) and a high genetic background scenario using a sewage sample. Both matrices were spiked with a suite of diverse viruses at known concentrations to evaluate viral particle recovery. Finally, three preamplification protocols were applied. One protocol targeting both DNA and RNA viruses, one targeting only DNA viruses, and one targeting only RNA viruses. The idea behind this comparison is that individual pipelines for DNA and RNA viruses should improve the sensitivity compared to protocols which simultaneously captures both genome types. Finally, the results were validated in unspiked sewage samples.

## Materials and Methods

Metaviromic analyses were conducted in spiked PBS and sewage samples, using different preamplification pipelines. The different the steps in workflow, from sample processing to bioinformatic analysis, are summarized in Fig. S1.

### Preparation of Virus Spiking Solutions

For spike-in experiments, individual virus spiking solutions were prepared for 14 viruses with diverse characteristics. A list of all viruses used, their taxonomic classification and their genome and capsid characteristics, as well as their hosts cells is given in Table S1. As species representatives for double-stranded DNA viruses (dsDNA), human mastadenovirus 2 (HAdV-C2, kindly donated by Rosina Girones, University of Barcelona and from now on referred to as HAdV), JC Polyomavirus (JCPyV MAD4 strain, ATCC VR-1583) and bacteriophage T4 (DSMZ 4505) were selected. As a representatives of single-stranded DNA viruses (ssDNA), adeno-associated virus 2 (AAV-2; kindly donated by Cornel Fraefel, University of Zurich) was used. Single-stranded RNA viruses (ssRNA) included MS2 bacteriophage (ATCC 15597-B1), enterovirus B (Echovirus 11 (E11) Gregory strain, ATCC® VR37TM, and from now on referred to as EV-B throughout the manuscript), Sendai virus (SeV; kindly provided by Dominique Garcin, University of Geneva) and human astrovirus MLB1 (HAstV MLB1; kindly donated by Susana Guix and Albert Bosch, University of Barcelona), human norovirus GII (NoVGII), hepatitis A virus (HAV) and murine norovirus type I (MNV) (all kindly donated by Anna Charlotte Schultz, Technical University of Denmark). For double-stranded RNA (dsRNA), mammalian orthoreovirus 1 (MRV-1 Lang Strain, ATCC® VR-230), rotavirus A (RoV-A; ATCC® VR-2018™) and Pseudomonas bacteriophage phi 6 (ɸ6, DSMZ 21518) were added.

Viruses were propagated on their respective bacterial host or mammalian cell line (Table S1). Bacteriophages were harvested after an overnight (o/n) incubation with the bacterial host, were centrifuged at 3000×*g* for 10 min to remove bacterial debris and were filtered through low-binding protein 0.45 µm and 0.22 µm Sterivex filters (Millipore, Massachusetts). Filtered solutions were preserved at 4 °C until further use.

Most mammalian viruses were propagated in-house. Propagation was performed in 125 cm^2^ cell culture flasks containing sub-confluent host cell monolayers using a Multiplicity of Infection (MOI) of 0.1 to 1 depending on the virus. After 1 h incubation at 37 °C with regular agitation, cells were washed with PBS (140 mM NaCl, 2.68 mM KCl, 10 mM Na2HPO4) (Cat. No. 18912014, thermofisher Scientific) to remove non-adhered viral particles. Exposed cells were incubated at 37 °C in a 5% CO_2_ atmosphere and were supplemented with DMEM at 2% of bovine serum for 5 to 10 days. Then viral particles were released from infected cells by three freeze–thaw cycles. Cell debris was removed by centrifugation at 3000×*g* for 15 min and the supernatant was filtered using low-binding protein 0.45 µm Sterivex filters (Millipore, Massachusetts). To prevent potential damage to viral capsids, the filtered solutions were preserved at 4 °C for a duration of one month before their subsequent use.

Four viruses, norovirus GII, SeV, AAV-2 and HAstV MLB1 were provided as stock solutions and were not further propagated. Specifically, NoV GII was received as a 2% fecal suspension from a clinical sample. To minimize companion microbiota present in the sample and reduce its genetic background, the fecal suspension was centrifuged at 3000×*g* for 15 min. The supernatant was filtered and stored as described above. SeV was received as a stock solution produced by propagation in embryonated eggs as described elsewhere (Strahle et al., 2006). AAV-2 was received as a stock solution produced in HEK293T using recombinant plasmids as described by (Samulski et al., 1987).

The virus concentration in each individual stock solution was determined by (RT)qPCR as described below. Stock solutions were then diluted with PBS to a virus particle concentration corresponding to approximately 1x10^5^ GC/mL. These solutions served as the final spiking solutions.

### Nuclease Treatment, Nucleic Acid Extraction and (RT)qPCR Analysis

Prior to nucleic acid (NA) extraction, viral stock solutions were nuclease treated to remove any free RNA or DNA. For each viral stock solution, 150 µl aliquots were treated with 30 U of TurboDNase (Ambion Cat. No. AM2238), 12U of Benzoase nuclease (Sigma Aldrich—E1014-5KU) and 5U of RNase A and 200U RNase T1 (Thermo Fisher Cat. No. AM2286) for 2 h at 37 °C. After nuclease treatment, NAs were extracted using the Qiagen Viral RNA Mini Kit (Cat. No. 22906, Qiagen, Valencia, CA, USA) without RNA carrier. NA were eluted using 60 μL of elution buffer.

(RT)qPCR analyses were conducted on tenfold dilutions of NA extracts in order to minimize the impact of inhibitors present in sewage samples. The different assays used are detailed in Table S2. For dsRNA and ssRNA viruses, quantifications were performed using the RNA Ultrasense One-Step Quantitative RT-PCR System kit (Invitrogen Cat.No.: 11732–927) by adding 5 µl of NA template. For DNA TaqMan assays, TaqMan Environmental PCR Master Mix kit (Applied Biosystems Cat. No. 4396838) was used by adding 10 µl of NA as template. Finally, a DNA SYBR green assay with TB Green™ Advantage® qPCR Premix kit (Takara, Cat. No. 639676) was used for bacteriophage T4 by adding 2.5 µl of NA as template. All (RT)qPCR reactions had a final volume of 25 μl and were run on a Mic qPCR Cycler device (Bio Molecular Systems). All (RT)qPCR runs included water as non-template controls (NTC). A negative extraction control (NEC) to discard cross-contamination during sample processing was also included. Calibration curves were generated using gblock standards (IDT, Coralville, IA, USA). These standards consisted of a minimum of 5 known concentrations that spanned the expected concentration range in the sample, were incorporated for each assay. For a comprehensive list of the standard concentrations, please refer to the supplementary section.

The PCR efficiencies, calibration curve slope and intercept of each assay is reported in Table S2, along with a checklist of experimental details as requested by the minimum information for publication of quantitative real-time PCR experiments (MIQE) guidelines in Table S3 (Bustin et al., 2009).

### Spiking of PBS and Sewage Sample and Concentration of Viral Particles

Spiking experiments were performed in a PBS and in raw sewage collected from the Vidy wastewater treatment plant (Lausanne, Switzerland). Sampling was approved by Le service de l’eau de la Ville de Lausanne, which manages the Vidy wastewater treatment plant. Aliquots of 105 mL were spiked with 1 mL of each of the virus spiking solutions to yield a final volume of 120 mL. We aimed for a spiked concentration of viral particles corresponding to 1 × 10^5^ GC per mL for each virus. This concentration was chosen as an acceptable starting point to detect spiked viruses by (RT)qPCR, assuming a LOD close to 50 GC per PCR reaction. The theoretical concentrations of spiked viruses are shown in Table 1. Spiked solutions were stirred for 1 h at room temperature. Then, 100 mL of the samples were mixed with 200 mL of 0.25 N glycine (Sigma Aldrich) at pH 9.5 for 30 min with constant rocking at 180 rpm on ice to facilitate the elution of viruses from organic particles. The sample was filtered through a 0.45 μm SteriCup filters (Merck, Cat. No. S2HVU02RE) and subsequently by a 0.22 μm SteriCup filters (Merck, Cat. No. SCGVU02RE). 50 mL of the filtrate was transferred to two different centrifugal filter units with a cut-off size of 10 kDa (Centricon Plus-70; Millipore Cat. No. UFC701008) and were centrifuged 30 min at 3000 *xg*. This step was repeated three times until the entire sample (300 mL) was processed. Viral concentrate was recovered by inverting the unit and centrifuge for 3 min at 1000 *xg* the centrifugal unit. Obtained viral concentrates (approximately 900 to 1000 µl) were collected and stored at − 20 °C until further use.

**Table 1:** Total amount of spiked viruses as genomic copies (GC) in low background sample (PBS) and high background sample (sewage)

	HAV	NoVGII	HAdV	EV-B	MNV	AAV-2	JCPyV	HAstV MLB1	MS2	RoV-A	SeV	Phi6	T4
Genome conformation	ssRNA +	ssRNA +	dsDNA	ssRNA +	ssRNA +	ssDNA	dsDNA	ssRNA +	ssRNA +	dsRNA	ssRNA-	dsRNA	dsDNA
Theoretical amount spiked (100 mL)	2.35 × 10^8^	7.83 × 10^6^	7.16 × 10^6^	9.68 × 10^7^	1.67 × 10^7^	2.57 × 10^7^	7.57 × 10^7^	2.43 × 10^7^	4.55 × 10^7^	3.20 × 10^7^	1.93 × 10^7^	3.29 × 10^7^	5.10 × 10^7^
Low background sample (PBS) (100 mL)	1.64 × 10^7^	3.58 × 10^6^	3.96 × 10^6^	2.45 × 10^6^	1.47 × 10^5^	2.04 × 10^6^	4.14 × 10^7^	7.03 × 10^4^	1.10 × 10^7^	3.18 × 10^7^	1.05 × 10^7^	2.40 × 10^6^	3.75 × 10^7^
Recovery (%)	7.0	45.6	55.2	2.5	0.9	7.9	54.6	0.3	24.3	99.5	54.3	7.3	73.6
High background sample (sewage) (100 mL)	8.17 × 10^5^	2.01 × 10^6^	1.32 × 10^6^	2.01 × 10^5^	4.48 × 10^4^	1.13 × 10^6^	1.68 × 10^8^	4.29 × 10^5^	4.97 × 10^6^	5.09 × 10^7^	9.33 × 10^6^	1.15 × 10^6^	1.96 × 10^7^
Recovery (%)	0.4	25.6	18.4	0.2	0.3	4.4	221.2	1.8	11.0	159	48.3	3.5	38.5

The table shows also the obtained recovery for each viral type. Quantifications by (RT)-qPCR refer to 100 mL sample

### Quantification of Virus Recovery

The concentrate was nuclease-treated and NA were extracted and enumerated by (RT)qPCR as detailed previously in Nuclease treatment, nucleic acid extraction and (RT)qPCR analysis section. Recovery was calculated as the ratio of spiked virus recovered after sample processing expressed in total genomic copies (GC) and the number of genomic copies (GC) spiked into the 120 mL PBS or sewage as expressed in Eq. 1. The GC per sample and GC added per mL of stock suspensions were calculated according to the formulas presented in Eq. 1 (A and B, respectively).

$$\text{GC in samples} \left(A\right)=\left({C}_{PCR} \times \frac{{V}_{extract}}{{V}_{PCR}}\right) \times 10\times \frac{{E}_{concentrate}}{{E}_{sample}}$$

$$\text{GC in stocks} \left(B\right)=\left({C}_{PCR}\times \frac{{V}_{Extract}}{{V}_{PCR}}\right)\times 10 \times \frac{1000}{S} \times {F }_{stock}\times \frac{{V}_{processed}}{{V}_{sample}}$$

$$Recovery (\%)=\frac{\text{measured GC in sample (A)} }{ \text{spiked virus GC added (B)}} \times 100$$

C_PCR_ is the template concentration (GC/reaction) determined by (RT)qPCR or qPCR, *V*_extract_ is the total volume of RNA extract (60 µl), V_PCR_ is the volume of extract analyzed by (RT)qPCR (5 µl for RNA, 10 µl for DNA and 2,5 µl for DNA SYBR Green assay), the factor 10 accounts for the tenfold dilutions of the original NA extracts, S accounts for the volume of the stock (140 µl for stocks), *E*_*sample*_ is the volume of the viral concentrate extracted (150 µl or 300 µl); *E*_*concentrate*_ is the total volume of the viral concentrate, *F*_*stock*_ is the fraction of the extracted sample that is composed by viral particles after nuclease treatment (0.78 for viral stocks), *V*_*sample*_ is the volume of PBS or wastewater used to concentrate viral particles (100 mL), *V*_*processed*_ is the total volume of PBS or sewage spiked (120 mL).

### Preamplification Protocols

**Fig. 1:**
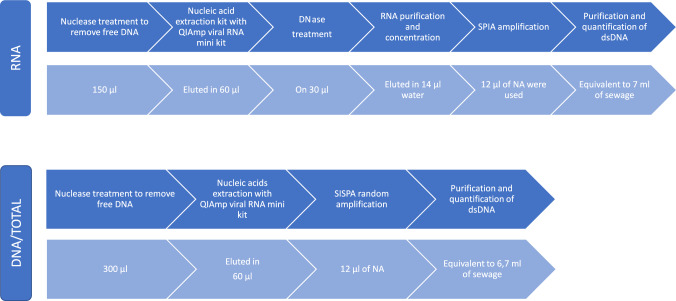
Summary of the different preamplification protocols tested. The different steps of the protocols are indicated in dark blue. The light blue field indicate the volumes used in each step and the corresponding raw sewage equivalent (colour figure online)

The remainder of the viral concentrates were used for metaviromic analysis. Each concentrate was subjected to three different preamplification protocols, which are summarized in Fig. 1A. In order to make the three protocols comparable, they were designed to capture a similar raw sewage equivalent (6.7 to 7.0 mL per preamplification reaction, see Fig. 1). This entailed those differing starting volumes of viral concentrate had to be used and reagent volumes were adjusted accordingly.

**Fig. 2:**
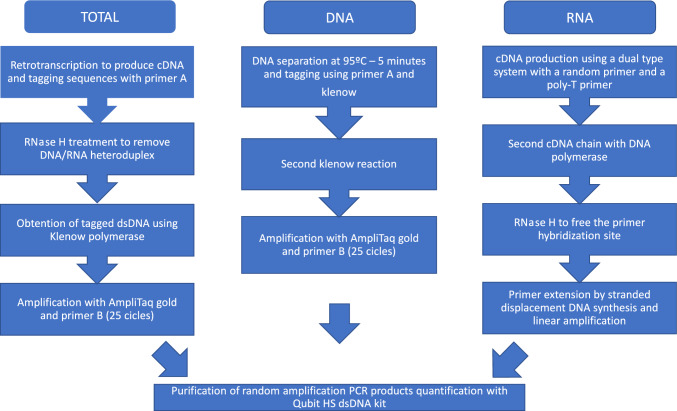
Detailed scheme with important steps involved in the preamplification for TOTAL, DNA and RNA pipelines (colour figure online)

In a first step, all viral concentrates were nuclease treated and NA was extracted as described in detailed in Nuclease treatment, nucleic acid extraction and (RT)qPCR analysis section. Subsequently, each viral extract was preamplified by three different protocols. All the three protocols were started with two independent reactions using 6 µl of NA. A diagram detailing the three procedures is presented in Fig. 2.

#### *TOTAL (DNA* + *RNA) Pipeline*

The TOTAL protocol (Fig. 2) is based on Sequence-Independent, Single-Primer Amplification (SISPA) and can capture both DNA and RNA viruses (Hjelmsø et al., 2017; Reyes & Kim, 1991; Wang et al., 2003). In brief, NAs were retrotranscribed using SuperScript III (Cat. No. 18080093, Life Technologies) and tagged using primer A (5′-GTTTCCCAGTCACGATANNNNNNNNN′-3). Hybrid DNA-RNA chains were treated with RNase H (Cat. No. 18,021–071, Thermofisher) to remove RNA and leave only the tagged cDNA. Immediately thereafter, a second cDNA strain was constructed using Exo Klenow polymerase (Cat. No. EP0421, Thermofisher). In order to obtain enough dsDNA as starting material for library preparation, a PCR amplification step with AmpliTaqGold (Cat. No.4311806, Life Technologies, Austin, Texas, USA) was performed, using the constant region present in primer A targeted by primer B (5′-GTTTCCCAGTCACGATA′-3). After 10 min at 95 °C to activate DNA polymerase, the following PCR program was used: 25 cycles of 30 s at 94 °C, 30 s at 40 °C, and 30 s at 50 °C with a final step of 60 s at 72 °C. Finally, dsDNA amplified products were purified and eluted in 15 µl of water using the Zymo kit clean and concentrator (D4013, Zymo research, USA).

#### DNA Pipeline

The DNA pipeline selectively targets DNA viruses and is based on the approach by Kramná & Cinek, (2018). The DNA protocol follows the same SISPA procedure as the TOTAL protocol, but omits the retrotranscription and RNase H steps (Fig. 2). Briefly, DNA strands were separated by heating at 95 °C for 5 min and subsequently chilled on ice to keep dsDNA strands separated. Immediately after, the separated strands were tagged using primer A and Exo Klenow polymerase. A second cycle of Klenow polymerase reaction was performed to ensure the capture of ssDNA viruses. After obtaining tagged dsDNA genomes, a PCR amplification step using primer B and AmpliTaqGold was performed as described in TOTAL (DNA + RNA) pipeline. Amplified dsDNA products were purified using the Zymo kit clean and concentration and eluted in 15 µl of water.

#### RNA Pipeline

RNA was selectively analyzed using a Single Primer Isothermal Amplification (SPIA) approach adapted from (Myrmel et al., 2017). For this protocol, the extracted NA were subjected to a second nuclease step, to remove any viral DNA obtained during NA extraction. Specifically, 30 µl of NA were DNase treated using 1 µl of TurboDNase (Ambion Cat. No. AM1907) for 20 min at 37 °C. The nuclease reaction was stopped by using DNase Inactivation Reagent included in the TurboDNase kit (Ambion Cat. No. AM1907) according to the manufacturer’s instructions. The nuclease-treated NA then underwent an additional purification and concentration step using the RNeasy MinElute Cleanup Kit (QIAgen, Cat. No.74204) and purified RNA was eluted in a final volume of 14 µl. cDNA was synthetized in two independent reactions using 6 µl of RNA as input with the Ovation RNA-Seq System V2 (NuGen, CA, USA; Part No. 7102) as detailed in the manufacturer’s manual. Briefly, cDNA was produced using a combination of random RNA and poly-T primers. A complementary chain for the cDNA was synthesized with a DNA polymerase. After second strand synthesis, RNA fragments of the SPIA primers were removed by using RNase H and DNA polymerase extended and isothermally amplified the tagged genomes. Amplification products from each of the SPIA reactions were pooled and purified as detailed in TOTAL (DNA + RNA) pipeline section.

### Quality Control, Library Preparation Sequencing

All purified dsDNA viral preparations were quantified using Qubit 2.0 (Cat. No. Q32854, Life Technologies, Oregon, USA) and the HS dsDNA kit (Cat. No. Q32851). Libraries were constructed using Nextera XT DNA sample preparation kit (Illumina Inc.) following manufacturer’s instructions. Samples were sequenced on two separate runs using Illumina MiSeq 2 × 300 with paired end reads.

### Bioinformatic Analysis

For the bioinformatic analysis, we utilized Genome detective, a web-based bioinformatics pipeline specifically designed to accurately and efficiently identify, assemble, and classify all known viruses present in viral metagenomics data (Vilsker et al., 2019). This platform, accessible at https://www.genomedetective.com/, offers a comprehensive solution for conducting robust viral analysis within viral metagenomics datasets. This bioinformatic pipeline involves several steps that are summarized below. Firstly, low-quality reads and adapters were trimmed using Trimmomatic (Bolger et al., 2014). This process ensured that only high-quality reads were retained for subsequent analysis, but duplicate reads were not removed. Identified candidate viral reads were selected using the protein-based alignment method DIAMOND (Buchfink et al., 2014) and non-viral sequences were discarded. Subsequently, viral paired-end reads were assembled with metaSPAdes (Nurk et al., 2017) and taxonomically classified with NCBI-BLASTX and NCBI-BLASTN against NCBI RefSeq viral database (Vilsker et al., 2019; Wheeler et al., 2007). The generated contigs are globally aligned against the closer reference genome included in the viral RefSeq to calculate the genome coverage (Vilsker et al., 2019).

The performance of the three preamplification protocols was evaluated by assessing the number of reads taxonomically assigned to the spiked viruses and their coverage in both the low (PBS) and high (sewage) genomic content.

In an attempt to type retrieve viral species of Enterovirus and Norovirus detected, assembled contigs were additionally analyzed using the RIVM Enterovirus (https://www.rivm.nl/mpf/typingtool/enterovirus/) and Norovirus (https://www.rivm.nl/mpf/typingtool/norovirus/) typing tools (Kroneman et al., 2011).

### Application of the Preamplification Protocols to Determine the Presence of Mammalian Viruses in Sewage

The performance of the preamplification protocols was assessed in unspiked sewage samples. Specifically, three 24-hour composite samples of raw sewage were collected from the Vidy wastewater treatment plant and were concentrated. The first sample (100 mL) was processed as described above. The other two samples had a starting volume of 300 mL, and they were concentrated in their entirety as described above, but without the amendment of the glycine solution. The glycine treatment was omitted to increase the sewage equivalents in the final sequencing reaction.

## Results

### (RT)qPCR Quantification and Recoveries of Spiked Viruses in PBS and Sewage

Viral quantifications and recoveries in both low genetic background sample (PBS) and high genetic background sample (Sewage) are presented in Table 1. Data to calculate the recovery for spiked viruses is provided in table S4. Data to generate the calibration standard curves is provided in table S5.

All NTCs and NECs were negative, whereas all spiked viral targets in both matrices fell within the range of the (RT)qPCR calibration curves. In PBS, the recoveries for spiked viruses ranged from a minimum of 0.3% for HAstV MLB1 to a maximum of 99.5% for RoV. In sewage, recoveries ranged from a minimum of 0.2% for EV to a maximum of 221.2% for JCPyV, respectively. Recoveries over 100%, as obtained for JCPyV and RoV, are indicative of a high background concentration of these viruses in sewage. For mammalian reovirus, recoveries over 100% were obtained in both PBS and sewage. Given that the negative NTCs and NECs indicated an absence of contamination, we attribute the high recovery to either a faulty quantification of the spiked virus concentration or a problem with the real-time assay employed. Due to the abnormal values retrieved, mammalian reovirus (RT)qPCR data was excluded from Table 1.

**Fig. 3:**
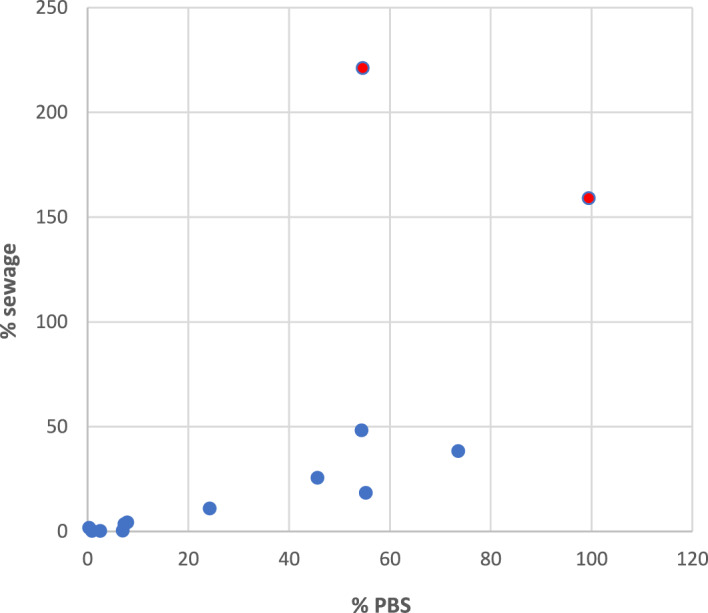
Comparison of the calculated recovery for all spiked viruses quantified by (RT)qPCR depending on the matrix tested. Presented data omitted mammalian orthoreoviruses as the calculated recovery presented unrealistic recoveries for both PBS and sewage. Plots the recovery (%) of spiked viruses in PBS (X-axis) vs sewage (Y-axis) including RoV and JCPyV (red dots). Higher recoveries for JCPyV and Rotaviruses, can be explained by their high natural presence in sewage. *R* = 0.67 (including JCPyV and RoVA); *R* = 0.91 (excluding JCPyV and RoVA)

Figure 3 compares the recovery for each virus in PBS and sewage (data given in Table S6). The recoveries of the viruses in PBS were positively correlated with the respective recoveries in sewage (*r* = 0.67). This correlation became even stronger when viruses with higher background concentrations in sewage (JCPyV and RoV) where excluded (*r* = 0.91).

### Spiked Viral Reads and Coverage Retrieved According to Preamplification Pipeline

A summary of the mammalian viral species detected in spiked PBS and sewage samples are provided in Tables 2 and 3, respectively. A list of detected mammalian viruses in unspiked sewage samples is provided in Table 4. A summary of the MiSeq output and statistics regarding the number of viral and non-viral sequences, the number of mapped reads and other run related and bioinformatic parameters are given in the Table S7. Additionally, an excel file containing a complete list of retrieved viral reads from spiked samples as well as unspiked sewage samples obtained with genome detective is presented in Tables S8 and S9.

**Table 2:** Reads and contigs taxonomically assigned to viral species in spiked PBS

Virus—Accession number		TOTAL			DNA			RNA		
		n.º Reads	Contigs	Coverage (%)	n.º Reads	Contigs	Coverage (%)	n.º Reads	Contigs	Coverage (%)
Non-segmented viruses										
JC polyomavirus (JCPyV)—NC_001699.1		83288	1	100	35586	1	100	273	9	66.6
Human mastadenovirus C (HAdV)—AC_000007.1		10578	2	99.9	4900	6	98.3	117	8	9.64
Bacteriophage T4—NC_0008644.4		13099	3	100	7540	129	90.5	–	–	–
Adeno-associated virus 2 (AAV-2)—NC_001401.2		247	4	90.8	38	2	37.7	–	–	–
Hepatovirus A (HAV)—NC_001489.1		–	–	–	–	–	–	1115051	1	99.8
Sendai virus (SeV)—NC_001552.1		–	–	–	–	–	–	243199	1	99.5
MS2 bacteriophage—NC_001417.2		–	–	–	–	–	–	536961	1	98.5
Enterovirus B (EV-B)—NC_038307.1		80	4	47.5	6	2	11	311263	1	100
Astrovirus MLB1 (HAstV MLB1)—NC_011400.1		–	–	–	–	–	–	995	4	96.5
Norwalk virus (NoVGII)—NC_008311.1		–	–	–	–	–	–	7622	1	95.3
Murine norovirus (MNV)—NC_008311.1		–	–	–	–	–	–	147	7	36.2
Segmented viruses										
Pseudomonas bacteriophage Phi6 NC_003714.1—NC_003715.1	Segment L	–	–	–	–	–	–	3343	1	98.9
	Segment M	–	–	–	–	–	–	898	1	95.9
	Segment S	–	–	–	–	–	–	677	1	98.1
Mamalian orthoreovirus (MRV) NC_013225.1—NC_013234.1	Segment L1	–	–	–	–	–	–	4144	1	99.9
	Segment L2	–	–	–	–	–	–	2335	1	100
	Segment L3	–	–	–	–	–	–	1803	1	98.1
	Segment M1	–	–	–	–	–	–	2320	1	98.0
	Segment M2	–	–	–	–	–	–	565	1	99.9
	Segment M3	–	–	–	–	–	–	1120	1	99.6
	Segment S1	–	–	–	–	–	–	679	1	77.7
	Segment S2	–	–	–	–	–	–	520	1	95.4
	Segment S3	–	–	–	–	–	–	647	1	94.2
	Segment S4	–	–	–	–	–	–	186	1	92.3
Rotavirus A (RoV-A) NC_011500.2—NC_011510.2	Segment 1	–	–	–	–	–	–	54406	1	99.9
	Segment 2	–	–	–	–	–	–	15606	1	99.0
	Segment 3	–	–	–	–	–	–	68663	1	96.8
	Segment 4	–	–	–	–	–	–	25714	1	99.9
	Segment 5	–	–	–	–	–	–	12877	1	87.9
	Segment 6	–	–	–	–	–	–	4024	1	99.0
	Segment 7	–	–	–	–	–	–	50445	1	86.2
	Segment 8	–	–	–	–	–	–	15086	1	99.8
	Segment 9	–	–	–	–	–	–	11772	1	99.7
	Segment 10	–	–	–	–	–	–	2012	1	76.2
	Segment 11	–	–	–	–	–	–	1048	1	98.5

The accession number used to stablish the genome coverage for each virus and segment are provided

**Table 3:** Reads and contigs taxonomically assigned to viral species in spiked sewage

Virus		TOTAL			DNA			RNA		
		n.º Reads	Contigs	Coverage (%)	n.º Reads	Contigs	Coverage (%)	n.º Reads	Contigs	Coverage (%)
Non-segmented viruses										
JC polyomavirus (JCPyV)—NC_001699.1		3791	1	100	1608	1	100	–	–	–
Human mastadenovirus C (HAdV)—AC_000007.1		51	14	18.3	54	10	14.9	–	–	–
Bacteriophage T4—NC_000866.4		108	26	6.9	150	22	7.3	–	–	–
Adeno-associated virus 2 (AAV-2)—NC_001401.2		2	1	7.4	–	–	–	–	–	–
Hepatovirus A (HAV)—NC_001489.1		–	–	–	–	–	–	115309	1	99.0
Sendai virus (SeV)—NC_001552.1		–	–	–	–	–	–	54732	1	98.7
MS2 bacteriophage—NC_001417.2		–	–	–	–	–	–	17583	1	100
Enterovirus B (EV-B)—NC_038307.1		–	–	–	–	–	–	7914	1	97.8
Astrovirus MLB1 (HAstV MLB1)—NC_011400.1		–	–	–	–	–	–	497	12	74.3
Norwalk virus (NoVGII)—NC_029646.1		–	–	–	–	–	–	189	6	23.2
Murine norovirus (MNV)—NC_008311.1		–	–	–	–	–	–	4	1	1.5
Segmented viruses										
Pseudomonas bacteriophage Phi6 NC_003714.1—NC_003716	Segment L	–	–	–	–	–	–	32	2	14.2
	Segment M	–	–	–	–	–	–	8	1	8.4
	Segment S	–	–	–	–	–	–	2	1	5.4
Mamalian orthoreovirus (MRV) NC_013225.1—NC_013234.1	Segment L1	–	–	–	–	–	–	38	1	19.0
	Segment L2	–	–	–	–	–	–	36	2	23.5
	Segment L3	–	–	–	–	–	–	8	1	3.3
	Segment M1	–	–	–	–	–	–	6	1	16.0
	Segment M2	–	–	–	–	–	–	–	-	-
	Segment M3	–	–	–	–	–	–	–	-	-
	Segment S1	–	–	–	–	–	–	1	1	7.8
	Segment S2	–	–	–	–	–	–	–	–	–
	Segment S3	–	–	–	–	–	–	–	–	–
	Segment S4	–	–	–	–	–	–	–	–	–
Rotavirus A (RoV-A) NC_011500.2—NC_011510.2	Segment 1	–	–	–	–	–	–	21781	2	89.5
	Segment 2	–	–	–	–	–	–	4214	1	90.0
	Segment 3	–	–	–	–	–	–	55097	1	94.8
	Segment 4	–	–	–	–	–	–	28312	1	64.8
	Segment 5	–	–	–	–	–	–	148	1	35.2
	Segment 6	–	–	–	–	–	–	1089	1	39.1
	Segment 7	–	–	–	–	–	–	32734	1	93.7
	Segment 8	–	–	–	–	–	–	2296	1	94.3
	Segment 9	–	–	–	–	–	–	2417	1	96.2
	Segment 10	–	–	–	–	–	–	155	1	53.3
	Segment 11	–	–	–	–	–	–	237	1	24.7

**Table 4:** Detailed list of mammalian viral species detected in unspiked sewage samples

Pipeline used		Total			DNA			RNA		
Viral Species		n.º reads (sample 1/2/3)	Contigs(sample 1/2/3)	Coverage (%) (sample 1/2/3)	n.º reads (sample 1/2/3)	Contigs (sample 1/2/3)	Coverage (%) (sample 1/2/3)	n.º reads (sample 1/2/3)	Contigs (sample 1/2/3)	Coverage (%) (sample 1/2/3)
human mastadenovirus F		4/–/–	2/–/–	2.4/–/–	–	–	–	–	–	–
Human polyomavirus 1		–/6/–	–/2/–	–/16.5/–	–	–	–			
Human polyomavirus 2		–/8/–	–/2/–	–/23.5/–	–	–	–	–	–	–
Human bocavirus 3		–/2/–	–/1/–	–/7.3/–	–/2/–	–/1/–	–/7.1/–	–	–	–
Adeno–associated virus—2		–/2/–	–/1/–	–/8.5/–	–/8/–	–/2/–	–/19.1/–	–	–	–
Enterovirus A		–	–	–	–	–	–	–/6/–	–/1/–	–/7.9/–
Enterovirus B		–/2/–	–/2/–	–/5.7/	–	–	–	24/84/3	1/2/2	3.6/6.3/9.2
Enterovirus C		–	–	–	–	–	–	654/100/16	6/9/4	43.3/50.2/26.2
Enterovirus D		–	–	–	–	–	–	–/–/4	–/–/1	–/–/5.6
Rhinovirus A		–	–	–	–	–	–	–/–/6	–/–/3	–/–/13.6
Rhinovirus B		–	–	–	–	–	–	–/–/4	–/–/2	–/–/11.1
Parechovirus A		–	–	–	–	–	–	–/–/13	–/–/4	–/–/23.5
Salivirus A		–	–	–	–	–	–	197/67/162	7/5/7	24/16.4/63.2
Aichi virus		–	–	–	–	–	–	1451/509/290	8/9/7	46.8/63/55.4
Cardiovirus B		–	–	–	–	–	–	31/34/–	4/2/–	14.4/9.5/–
Cosavirus E		–	–	–	–	–	–	–/6/–	–/1/–	–/5.8/–
Cosavirus A		–	–	–	–	–	–	–/6/–	–/1/–	–/4.6/–
Rabbit kobuvirus		–	–	–	–	–	–	–/6/–	–/1/–	–/4.7/–
Norovirus GI		–	–	–	–	–	–	–/11/156	–/3/5	–/10.6/75
Norovirus GII		–	–	–	–	–	–	–/65/35	–/6/7	–/34.4/41.7
Norwalk virus		–	–	–	–	–	–	6/–/–	1/–/–	3.6/–/–
Sapporo virus		–	–	–	–	–	–	27/24/25	2/2/4	5.2/8.2/30.5
Mamastrovirus 1		–	–	–	–	–	–	–/2038/–	–/2/–	–/91.7/–
Mamastrovirus		–	–	–	–	–	–	1210/–/–	10/–/–	61.9/–/–
Mamastrovirus 2		–	–	–	–	–	–	–/–/4	–/–/1	–/–/5.7
Mamastrovirus 9		–	–	–	–	–	–	–/20/–	–/3/–	–/19.1/–
Astrovirus MLB1		–	–	–	–	–	–	13/58/38	2/5/4	3.5/40.3/47.3
Astrovirus MLB2		–	–	–	–	–	–	33/–/4	3/–/1	8.9/–/6.7
HMO astrovirus A		–	–	–	–	–	–	–/132/218	–/6/4	–/55.8/80.7
Astrovirus VA3		–	–	–	–	–	–	–/6/22	–/1/3	–/6.2/31.5
Bastrovirus		–	–	–	–	–	–	–/28/–	–/3/–	–/11.9/–
Chicken astrovirus		–	–	–	–	–	–	–/10/–	–/1/–	–/5.1/–
Rotavirus A	–	–	–	–	–	–	–	200/–/–	2/–/–	23.3/–/–
	–	–	–	–	–	–	–	77/–/–	2/–/–	25.4/–/–
	–	–	–	–	–	–	–	148/–/–	1/–/–	14.1/–/–
	–	–	–	–	–	–	–	121/–/–	1/–/–	40.6/–/–
	–	–	–	–	–	–	–	179/–/–	1/–/–	32.3/–/–
	–	–	–	–	–	–	–	8/–/–	1/–/–	28.9/–/–

The character “/” is used to delimit results from three different sewage samples tested. For each sample, only the best match for a given species is indicated. More information about the detected species and each of the samples is given in Supplementary Table 9

Despite attempts to remove bacteria and free DNA by nuclease treatment, a high percentage of reads were taxonomically classified as non-viral (Table S7). In the PBS sample, which contained only the spiked viruses, both TOTAL and DNA pipelines retrieved 76 and 74% non-viral reads, respectively. For RNA, the number of non-viral reads retrieved was only 14% in spiked PBS. In spiked sewage, the number of non-viral reads was 69 and 70% for TOTAL and DNA pipelines and 59% for the RNA pipeline.

**Fig. 4:**
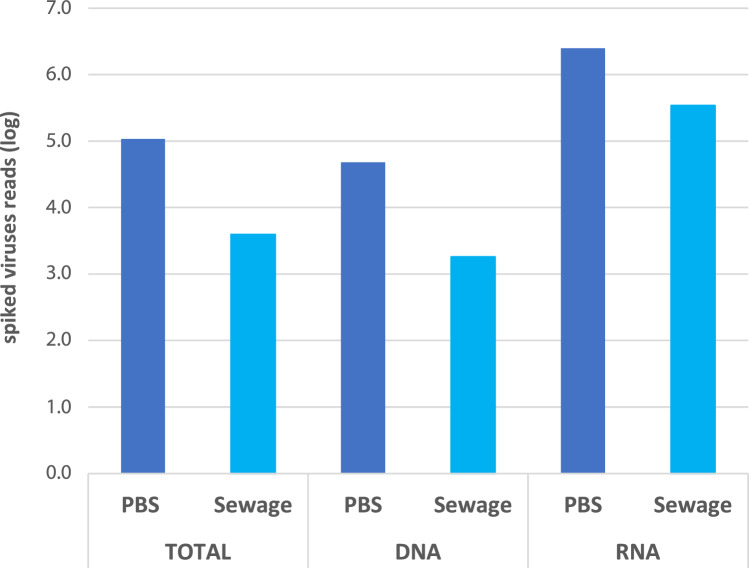
Number of total reads associated with the spiked viruses in each of the three preamplification protocols, in spiked PBS and spiked sewage

The number of total reads associated with the spiked viruses are plotted in Fig. 4. Data to plot Fig. 4 is presented in Table S10. The RNA pipeline yielded the highest number of reads in both matrices studied, whereas the DNA pipeline yielded the lowest number. Given that most of the spiked viral species had an RNA genome, this discrepancy is not surprising. The TOTAL pipeline captured a lower number of reads compared to the RNA pipeline, indicating that it was less efficient at capturing RNA viruses. The observed suboptimal performance of the TOTAL pipeline may be attributed to potential inhibition of the reverse transcriptase enzyme during the cDNA production process, though this remains to be confirmed in future work.

The specific number of reads for each spiked virus species, along with the genome coverage obtained, is listed in Tables 2 and 3. For the DNA pipeline, the maximum genome coverage obtained was 100% (JCPyV) in both sewage and PBS. The lowest coverage in PBS was 37% for AAV-2, whereas in sewage, no reads taxonomically assigned to this virus were identified. As expected, the DNA pipeline did not detect any RNA viruses. In the TOTAL pipeline with PBS, genome coverage ranged from 100% (JCPyV) to as low as 7.0% (T4 bacteriophage). Surprisingly, the TOTAL approach performed very poorly for RNA with only Enterovirus B being detectable in PBS but not in sewage. In the RNA pipeline, genome coverage in PBS ranged from a maximum of 100% (EV) to a minimum of 36.2% (MNV). In sewage, the coverage ranged from a maximum of 100% for MS2 bacteriophage to a minimum of 1.5% (MNV). This low genome coverage for MNV is in line with its low recovery (0.23%) by (RT)qPCR. For the segmented dsRNA viruses (Pseudomonas bacteriophage Phi6, RoV-A and MRV), all segments were found in PBS while in sewage 5 out of 11 segments of MRV were undetectable.

**Fig. 5:**
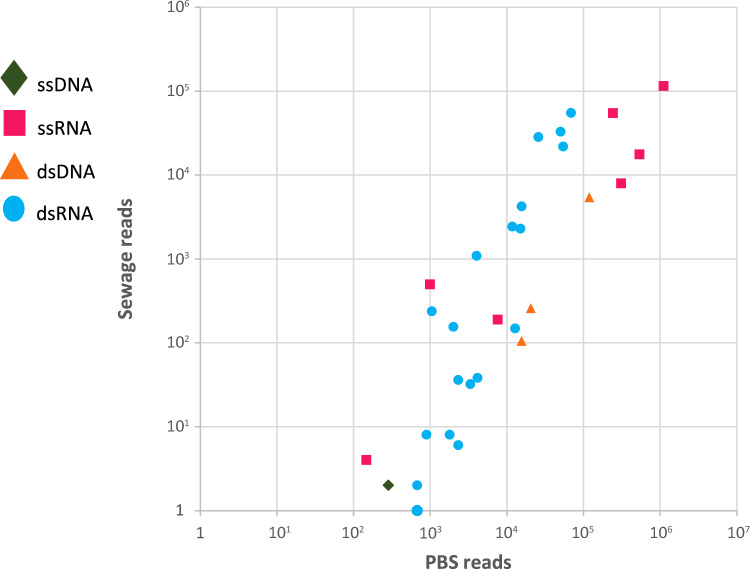
Number of reads in PBS (a low background genomic content) vs sewage (a high background genomic content) for all viruses and segments detected for all pipelines (TOTAL, DNA and RNA). Each symbol represents a virus or viral segment. Each possible genomic conformation is presented in a different color and shape.

We also correlated the number of reads retrieved for each viral species or genome segment in PBS against the corresponding reads in sewage (Fig. 5). A correlation coefficient of r = 0.81 was found when data from all the three preamplification protocols were used. When only the RNA preamplification was considered, r was still 0.81. However, sewage systematically yielded fewer reads compared to PBS, up to 3 orders of magnitude. Data used to calculate the correlation coefficients are provided in Table S11.

### Relation Between the Number of Reads Detected by Next Generation Sequencing (NGS) and (RT)qPCR Quantifications

**Fig. 6:**
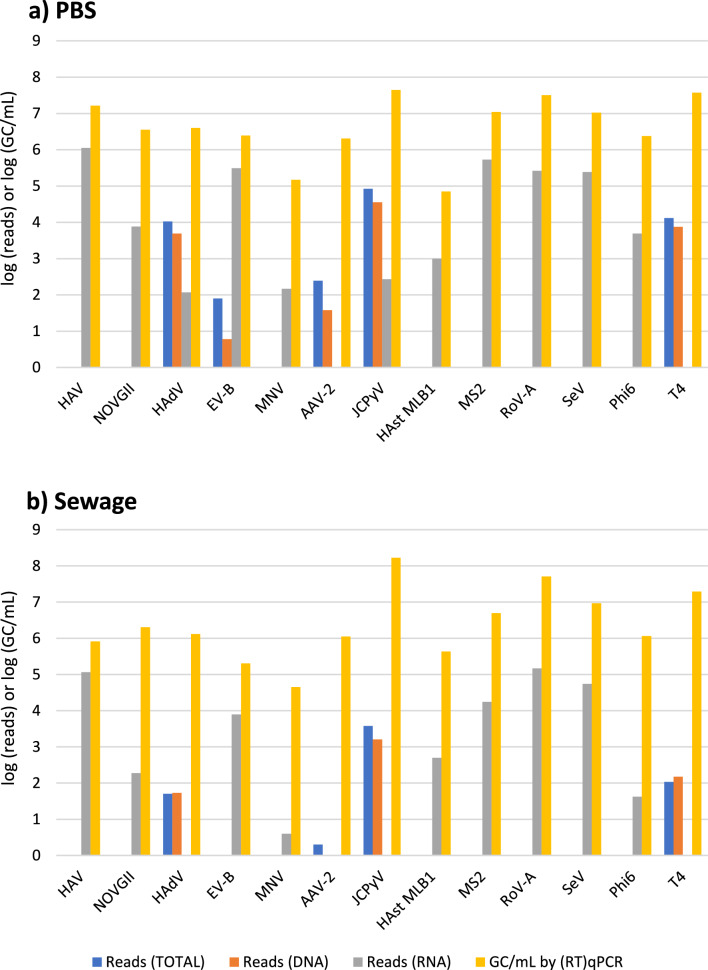
Stacked barplots comparing the log (RT)qPCR expressed as GC/mL with the log number of reads retrieved for PBS (**a**) and Sewage (**b**)

To determine if the number of reads associated with a given virus is representative of its concentration, we correlated the Reads Per Kilobase Million (RPKM) of each virus against the corresponding genome copies measured by (RT)qPCR. This analysis was performed separately for spiked PBS and sewage, and individually for each pipeline, whereby only DNA viruses were considered in the DNA and TOTAL pipelines, and only RNA viruses in the RNA pipeline. Furthermore, given the much larger spread of the NGS data compared to the (RT)qPCR data, this analysis was conducted on a log–log scale. A positive correlation could be found between the RPKM and the GC measured by each pipeline for each matrix considered (*r* ranged from 0.55 to 0.82 in PBS and from 0.62 to 0.82 in sewage, Table S12). High virus concentrations are thus associated with high viral reads (Fig. 6).

### Viral Species with Putative Pathogenic Potential in Unspiked Sewage Samples

**Fig. 7:**
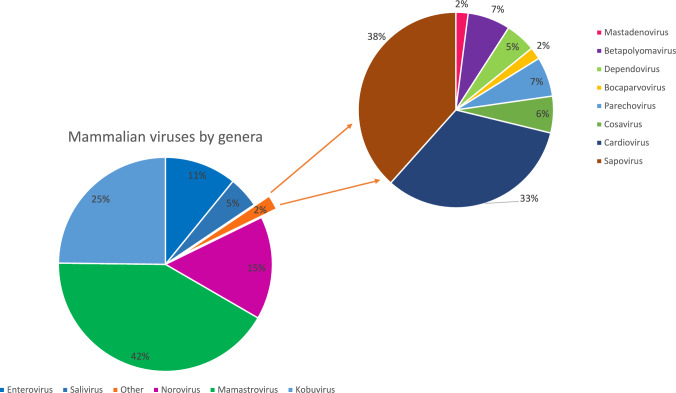
Detected genera in all the unspiked sewage samples, including the three different preamplification methods

A complete inventory of retrieved viral reads from unspiked sewage obtained with genome detective is presented in Table S9. The three preamplification protocols were applied to three unspiked sewage samples to detect the presence of viruses with potential to cause human disease and/or infecting mammalian hosts. As for the spiked samples, the number of non-viral reads in the unspiked samples was high in all of the preamplification protocol used (Table S7). A detailed list of all the viral species identified according to the preamplification protocol used, as well as their genomic coverage is given in Table 4. Detected families which contain human pathogenic viruses include members of the families *Picornaviridae, Caliciviridae, Astroviridae, Reoviridae, Adenoviridae, Polyomaviridae and Parvoviridae* and are presented in Fig. 7.

The TOTAL protocol identified viral reads belonging to the families *Adenoviridae, Polyomaviridae* (BKPyV and JCPyV) and *Picornaviridae*. However, the number of reads and the coverage retrieved for *Adenoviridae* and *Picornaviridae* contigs did not allow a proper typing of these viral species. In addition, enteroviruses were only detected in one of the three samples. Obtained results suggests a similar problem with RNA in the TOTAL pipeline as observed for the spiked experiment. DNA preparation identified members of the *Parvoviridae* family but no other any viral pathogens.

The RNA protocol yielded members of the families *Reoviridae*, *Picornaviridae*, *Astroviridae* and *Caliciviridae.* For HAstV MLB1, the coverage was as high as 91.7%, retrieving almost it’s full genome. In addition, several genera within the *Picornviridae* family were identified, including *Enterovirus* (EV species C and Rhinovirus A and B), *Salivirus, Kobuviru*s and *Cardiovirus*. For the *Caliciviridae* family, important pathogens such as NoVGI and NoVGII as well as sapoviruses were detected. In the case of NoVGI and NoVGII the genome coverage for one of the samples was high enough to enable viral typing of NoVGI, identifying a NoVGI.P1 | GI.9. Other retrieved contigs of NoVGII did not fully cover the ORF1/ORF2 junction and could not be typed.

Within *Reoviridae*, up to 6 different rotavirus A segments could be identified in one of the studied samples. However, none of the segments belonged to the VP4 region and only a partial sequence of VP7 was retrieved (Rotavirus A strain RVA/Human-wt/RUS/NN1918-16/2016/G1P8; Accession number: MN337577, sharing 99.0% nucleotide identity over 100% coverage).

Among non-human virus sequences, the DNA protocol detected mostly head–tail bacteriophages from the previously classified families *Podoviridae*, *Myoviridae* and *Siphoviridae* and some unclassified ssDNA circular viruses. Recently, these families have been abolished and reclassified into 22 different viral families as detailed in Turner et al., 2023. Finally, the RNA pipeline was dominated by phytoviruses from the families *Virgaviridae, Tombusviridae, Alphaflexiviridae* and *Tymoviridae*. Sequences related to unclassified picorna-like viruses and some insect viruses from the family *Dicistroviridae* were detected in all samples with some mammalian viruses detected sporadically. The predominant viral phage family was *Leviviridae.*

## Discussion

### High Genetic Background Reduces the Sensitivity of Viral Metagenomics of dsDNA and dsRNA Viruses

Three different protocols to preamplify nucleic acids before library preparation and NGS were tested in PBS and sewage spiked with several viruses at a known concentration. Across all virus species, the number of associated reads was higher in the PBS sample, which has a low genetic background, compared to the sewage sample with a high genetic background. The lower number of retrieved reads in sewage associated with the spiked viruses resulted in a reduced coverage in this matrix. The difference in the number of reads between the two matrices was more pronounced for double-stranded viruses (dsRNA and dsDNA) when compared to single-stranded viruses (ssRNA), if indigenous viruses in sewage (JCPyV and RoV-A) are excluded. The difficulty in capturing double-stranded RNA genomes may be due to an incomplete denaturation and tagging of their chains during cDNA production. Despite the high prevalence and viral loads of dsRNA viruses such as rotaviruses in sewage measured by (RT)qPCR they are rarely detected by NGS (Wang et al., 2020), with rare exceptions in viral metagenomics studies that use enrichment approaches (Martínez-Puchol et al., 2020; Strubbia et al., 2019) or a different matrix like sludge (Bibby & Peccia, 2013). Difficulties in sequencing dsRNA genomes have been highlighted and addressed by some authors by developing specific protocols to increase the reads associated to this particular conformation (Wilcox et al., 2019). Similarly, this issue might affect negatively double-stranded DNA conformations, which have been detected by qPCR in sewage but unable to be detected by using a viral metagenomics approach (Fernandez-Cassi et al., 2018).

### An association Exists Between Virus Recovery in Sewage and PBS and Between Virus Concentration and RPKM

To assess virus recovery, 14 different viruses were spiked at approximately the same concentration both in Sewage and PBS samples. Recoveries of spiked viruses were assessed by using specific (RT)qPCR. For most of the viruses tested, recoveries during sample concentration were somewhat higher in PBS compared to sewage, yet recoveries in the two matrices were positively correlated. Lower recoveries in sewage were expected due adsorption of viruses to particulate matter and subsequent losses during sample filtration steps (Gutierrez & Nguyen, 2012). Furthermore, higher quantifications of in PBS is expected by (RT)qPCR, due to the absence of inhibitory substances, which are abundant in sewage samples (Schrader et al., 2012). Only two spiked viruses, namely JCPyV and RoV-A, exhibited a higher concentration in sewage compared to PBS, resulting in recoveries over 100%. These viruses are commonly detected in high concentrations in raw sewage (Rusiñol et al., 2015; Wang et al., 2020). Both viruses were detected using (RT)qPCR on a different aliquot of the sample, presenting 2.32x10^7^ GC/L for RoV-A as published in Li et al., (2023) hence recoveries over 100% are not unexpected.

Despite EV-B and HAV having a very low (< 0.5%) recovery in sewage when quantified by (RT)qPCR, we retrieved a high number of reads and nearly complete coverage of the genome. In addition, in each matrix, a positive correlation between the log transformed (RT)qPCR and RPKM for each spiked virus and pipeline were found, indicating that sequencing can be used in a semiquantitative way. The preamplification procedure to achieve enough DNA for library preparation unavoidably introduces a bias in the relative abundance of the original sample which depends on the number of amplification cycles or the duration of the amplification cycle in isothermal methods (Regnault et al., 2021; Wang et al., 2023). This bias might be conditioned by other factors such as genome size of the virus or its GC% composition (Cremers et al., 2018).

Other studies have highlighted the difficulty to draw quantitative conclusion from metaviromic studies applied in the context of sewage sequencing (Hjelmsø et al., 2017; Wang et al., 2020). In addition, it has been reported the existing difficulties to detect by NGS means viruses that were highly abundant by (RT)qPCR (Bibby & Peccia, 2013; Fernandez-Cassi et al., 2018). Other factors besides the number of genomic copies such as genomic secondary structures might play a role in the ability to detect viral species by NGS (Price & Garhyan, 2017).

### Virus Selective Sample Preparation do Not Suppress Non-viral Reads

We made a concerted effort to enrich the samples in viruses while removing bacteria. Specifically, we used a gentle bacterial removal procedure (disposable stericup filters) to avoid bacterial membrane disruption and applied nuclease treatment prior to viral genome extraction to remove free genetic material. Nevertheless, a high proportion of reads were taxonomically classified as non-viral (from 59 to 76% of the total reads), in line with values reported in previous studies (Cantalupo et al., 2011; Fernandez-Cassi et al., 2018; Hjelmsø et al., 2019). Poor performance of negative selection methods to reduce bacterial contamination have been previously reported in the context of viral metagenomics (Hall et al., 2014). The presence of contaminants is a common issue on viromics studies, where these contaminants can be introduced at any point from stock production to subsequent concentration, amplification and sequencing steps as reviewed by Jurasz et al., (2021). These contaminants are inherent and unique in any processing pipeline, associated to sample processing, nucleic acids extraction and library preparation (Asplund et al., 2019). Our hypothesis is that the DNA pipeline is more sensitive to capture these contaminants in comparison to the TOTAL pipeline. Despite this lower efficiency, an important part of these sequences could be incorrectly considered as bacterial due to the high homology between phages and their hosts or viral sequences yet to be characterized also known as viral dark matter (Santiago-Rodriguez & Hollister, 2022). Better bioinformatic tools not limited by alignment methodologies and the lower diversity within viral databases are needed, especially in the context of environmental studies (Krishnamurthy & Wang, 2017). Further research is needed to improve the selective amplification of viral targets in complex matrices.

### Specific RNA Amplification in Unspiked Sewage Samples Improves the Detection of Mammalian RNA Viruses

The application of the three tested protocols to three different unspiked sewage samples showed an improved performance for the RNA pipeline compared to the TOTAL one to identify viruses within a genus that harbor viral species with pathogenic potential. The fact that this preamplification method contains a post-extraction DNase step might increase the removal of important dsDNA bacteriophages that are predominant in sewage. In unspiked sewage samples, the RNA pipeline based on a SPIA amplification detected viral reads related to mammalian viruses. Important families with pathogenic potential detected in the analyzed samples include *Reoviridae, Picornaviridae, Astroviridae and Caliciviridae* members. Some of the retrieved viral species had enough genome coverage to allow viral typing and sporadically, it was possible to retrieve almost their full genome. However, the low relative abundance of mammalian viruses in proportion with phytoviruses produced non-overlapping contigs of mammalian viruses, limiting the capacity of an appropriate typing. Interestingly, several reads related to human rotavirus segments were detected in one of the unspiked samples. This could be indicating that this pipeline is capable of tagging dsRNA genomes.

### Specific DNA Amplification in Unspiked Sewage Samples Does Not Improve the Detection of Mammalian DNA Viruses

An inferior performance of the DNA compared to the TOTAL pipeline was observed in the unspiked sewage samples. Here, the only genus containing viruses with a putative pathogenic potential identified by the DNA pipeline was a species from the *Parvoviridae* family, whereas the TOTAL pipeline identified members of *Adenoviridae* and *Polyomaviridae*. Aiming to solely amplify DNA viruses did not improve the detection of mammalian viruses neither in the unspiked sewage samples nor the spiked ones when compared to the TOTAL pipeline. The underperformance of the DNA pipeline may be rationalized by a more efficient tagging of the dsDNA bacteriophages from the class Caudoviricetes which are predominant in viral metagenomes from environmental samples including sewage (Cantalupo et al., 2011; Fernandez-Cassi et al., 2018; Ng et al., 2012). In addition, this pipeline included a denaturation step at 95 °C which aimed to more efficiently tag mammalian dsDNA viruses, but also let to more efficient tagging of indigenous head–tail dsDNA bacteriophages.

Consequently, the abundance of mammalian dsDNA viruses may be further biased and reduced by the subsequent PCR amplification (Gutierrez & Nguyen, 2012; Karlsson et al., 2013) and the limited output capacity of the MiSeqform platform. Other alternatives to preamplify viral genomes should be pursued to specifically amplify DNA viruses (e.g., Multiple displacement amplification (MDA) amplification).

### Strengths and Limitations of the Current Study

An important limitation of the study is the number of samples processed. A higher number of processed samples, spiked PBS and sewage and unspiked sewage, using the three described pipelines would provide more robustness to the results presented in the current study. Studies with a similar approach but with a higher number of samples should be pursued to confirm or discard the relationship between (RT)qPCR and the retrieved results by NGS. Recently, research conducted by Crossette et al., (2021) and Langenfeld et al., (2022) have used spike-in approaches for quantifying gene concentrations, including Antibiotic Resistant Genes (ARGs), marine phages and human DNA viruses such as polyomavirus, papillomavirus, adenovirus, in environmental samples to prove the potential of quantitative metagenomics. Unfortunately, these studies were focused on DNA targets, avoiding the inclusion of RNA standards. Furthermore, it is imperative that these studies encompass viruses with larger genome sizes, such as Herpesvirus or Poxvirus, to obtain a more comprehensive understanding. In this work, viruses with larger genome sizes (e.g., HAdV, RoV, SeV, T4; see Table S12) tended to yield a greater number of reads in sewage (Fig. 6). In this sense, future research following a similar approach but including move varied genome sizes needs to be conducted.

The preamplification methods have proven to present inherent biases as reported by Roux et al., (2016), which showed a preference of MDA for ssDNA viruses. Despite being widely used, SISPA has been associated with biases too and it’s not recommended for the study of RNA viruses according to López-Labrador et al., (2021). These biases do not only affect the preamplification phase but also, they can be linked to the library preparation (Pérez-Cataluña et al., 2021), the impact of the bioinformatic pipeline selected (Sutton et al., 2019) or the sequencing technology employed (de Vries et al., 2021).

Given the various biases involved in preparing viromic samples, it is necessary to establish comprehensive protocols that assess multiple variables throughout both the wet laboratory procedures and the subsequent data and bioinformatic analyses. To address this issue, a methodology similar to the studies conducted by Conceição-Neto et al., (2015, 2018) or Regnault et al., (2021) should be employed, wherein a systematic examination of the viral losses occurring during the wet lab processing is conducted specifically within the context of sewage viral metagenomics. These protocols should be validated using high-quality reference materials that encompass a range of viral genome conformations, lengths, and genome content, as outlined by Santiago-Rodriguez and Hollister, (2020).

A problem with cDNA production during the retrotranscription step in the TOTAL pipeline cannot be ruled out, as very few RNA viruses were detected in both the spiked sample and the native unspiked sewage samples. To identify issues during the retrotranscription step, it would be beneficial to include an RNA process control such as mengovirus, as recommended by Van Borm et al. (2020). This measure would help in pinpointing performance issues, including potential inhibition during cDNA production by retrotranscriptase. In addition, previous studies conducted in the field have indicated that the inaccurate termination of RNases may lead to the loss of RNA process controls that were spiked into the samples (Adriaenssens et al., 2018). This loss could potentially arise from an incompatibility in the inactivation of various nucleases. We acknowledge this possibility, which could not be definitively ruled out in the current study.

## Conclusions

1. We developed a RNA pipeline based on SPIA to study RNA viruses in sewage. The developed protocol provided good coverage for RNA viruses both in spiked PBS and sewage, allowing the detection of all RNA viruses spiked. Its application in unspiked sewage samples allowed the detection of putatively pathogenic RNA viruses. This method could also be used as a tool to perform ecological studies on the RNA virome from sewage.
2. The specific preamplification for DNA viruses didn’t increase the detection of mammalian viruses neither in the spiked samples nor the unspiked sewage samples. Alternative metaviromic preamplification protocols to increase the sensitivity for DNA animal viruses in sewage are needed.
3. Recoveries of the spiked viruses were higher in PBS for all viruses except for those viruses naturally occurring in Sewage used for the experiment. A log–log correlation between the (RT)qPCR quantifications in both matrices and the Reads Per Kilobase Million (RPKM) was found.
4. To implements sewage viral metagenomics as a surveillance tool for epidemiological purposes, positive selection methods such as post-library enrichment panels targeting viral families with pathologic potential are recommended to increase the coverage and sensitivity. This seems particularly relevant for DNA viruses for which specific amplification of ssDNA and DNA seems to favor viral phages.

## Supplementary Information

Below is the link to the electronic supplementary material.

Supplementary file1 (PDF 383 KB)

Supplementary file2 (DOCX 28 KB)

Supplementary file3 (DOCX 33 KB)

Supplementary file4 (PDF 115 KB)

Supplementary file5 (XLSX 14 KB)

Supplementary file6 (XLSX 13 KB)

Supplementary file7 (XLSX 11 KB)

Supplementary file8 (DOCX 17 KB)

Supplementary file9 (XLSX 1675 KB)

Supplementary file10 (XLSX 3732 KB)

Supplementary file11 (XLSX 16 KB)

Supplementary file12 (XLSX 19 KB)

Supplementary file13 (XLSX 17 KB)

## Data Availability

The complete sequencing data are available at https://www.ncbi.nlm.nih.gov/bioproject/948647 with the BioSample accessions numbers SAMN33911627, SAMN33911628, SAMN33911629, SAMN33911630 and SAMN33911631 (SRA SRR23986800—SRR23986814).
