# Supplementary figures and images for: Comparison of Three Viral Nucleic Acid Preamplification Pipelines for Sewage Viral Metagenomics
Source: Food Environ Virol. 2024 Apr 22;16(3):1–22. doi: 10.1007/s12560-024-09594-3 (PMC11422458; doi:10.1007/s12560-024-09594-3)

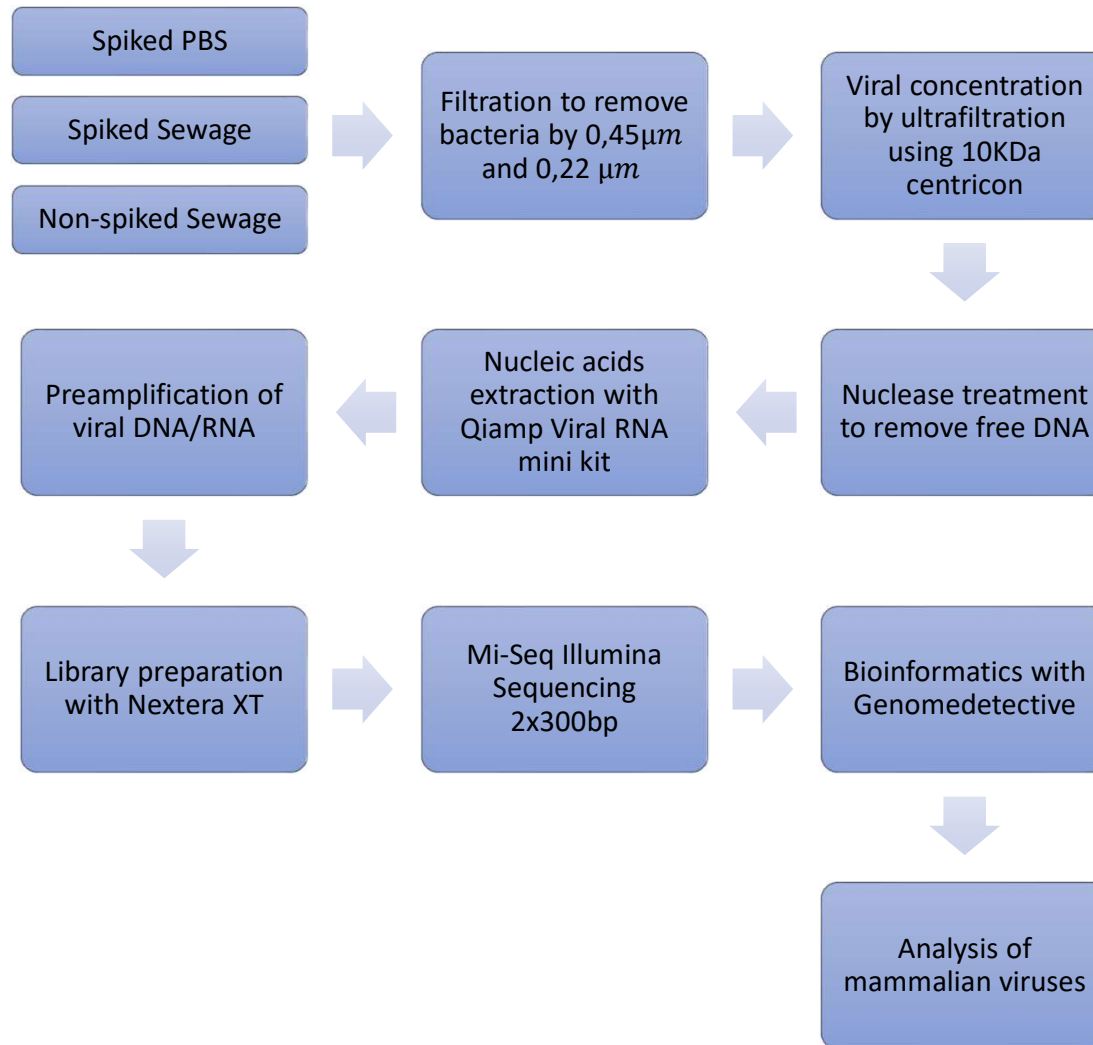

Supplement: Supplementary file 1 — Supplementary file1 (PDF 383 KB) [file 12560_2024_9594_MOESM1_ESM.pdf]
