# Supplementary material for: Comparison of Three Viral Nucleic Acid Preamplification Pipelines for Sewage Viral Metagenomics
Source: Food Environ Virol. 2024 Apr 22;16(3):1–22. doi: 10.1007/s12560-024-09594-3 (PMC11422458; doi:10.1007/s12560-024-09594-3)
Supplement: Supplementary file 2 — Supplementary file2 (DOCX 28 KB) [file 12560_2024_9594_MOESM2_ESM.docx]

| Virus Family | Viral species | Genome type | Genome Size  (Kbs)  Segmented (Y/N) | Envelope | Propagation host | Source |
| --- | --- | --- | --- | --- | --- | --- |
| *Picornaviridae* | Echovirus type-11  (EV-B) | ssRNA(+)  linear | 7,5 (N) | N | BGMK | Gregory strain, ATCC® VR37TM |
|  | Hepatovirus A (HAV) | ssRNA(+)  linear | 7,5 (N) | N | VERO | Kindly donated by Anna Charlotte Schultz, Technical University of Denmark (DTU) |
| *Caliciviridae* | Norovirus GII  (NoVGII) | ssRNA(+)  linear | 7,5 (N) | N | Fecal clinical sample  Not propagated* | Kindly donated by Anna Charlotte Schultz, Technical University of Denmark (DTU) |
|  | Murine Norovirus  (MNV) | ssRNA(+)  linear | 7,5 (N) | N | RAW  Not propagated* | MNV: Kindly donated by Anna Charlotte Schultz, Technical University of Denmark (DTU)  RAW: kindly donated by Beat Schwaller, University of Fribourg |
| *Astroviridae* | Astrovirus MLB1  (HAstV MLB1) | ssRNA(+)  linear | 6,1 (N) | N | Not propagated* | Kindly donated by Prof. Albert Bosch, University of Barcelona |
| *Leviviridae* | MS2 bacteriophage | ssRNA(+)  linear | 3,5 (N) | N | *E. coli* DSMZ 5695 | DSMZ 13767 |
| *Paramyxoviridae* | Murine respirovirus – Sendai virus (SeV) | ssRNA (-)  linear | 15,3 (N) | Y | Embryonated eggs  Not propagated* | Kindly donated by Dominique Garcin, University of Geneva |
| *Adenoviridae* | Human mastadenovirus 2 (HAdV-C2) | dsDNA  linear | 35 (N) | N | A549 | HAdV-C2: Kindly donated by Prof. Rosina Girones, University of Barcelona |
| *Polyomaviridae* | JC Polyomavirus  (JCPyV) | dsDNA  Circular | 5 (N) | N | COS-7 | ATCC VR-1583 - MAD4 strain  COS-7: Kindly donated by Sulliana Manley, Ecole Polytechnique Federale de Lausanne |
| *Myoviridae* | Bacteriophage T4 | dsDNA  linear | 169 (N) | N | *E.Coli* DSMZ 13127 | Escherichia coli bacteriophage T4 (ATCC 11303-B4)  Bacteriophages DSM 4505 |
| *Parvoviridae* | Adeno-associated virus type 2 (AAV-2) | ssDNA  linear | 4,6 (N) | N | HEK293T + helper plasmids | Kindly donated by Professor Cornel Fraefel, University of Zurich |
| *Reoviridae* | Mammalian  orthoreovirus 1 (MRV) | dsRNA  linear | 23,5 (Y, 10 segments) | N | L929 | ATCC® VR-230, Lang Strain |
|  | Human Rotavirus A (RoV-A) | dsRNA  linear | 18,5 (Y, 11 segments) | N | MA-104 | ATCC® VR-2018 |
| *Cystoviridae* | *Pseudomonas b*acteriophage phi 6 | dsRNA  linear | 13.5 (Y, 3 segments) | Y | *P. syringae* (DSMZ 21482) | DSM 21518  Kindly donated by Tim Julian, EAWAG |
