# Supplementary material for: Comparison of Three Viral Nucleic Acid Preamplification Pipelines for Sewage Viral Metagenomics
Source: Food Environ Virol. 2024 Apr 22;16(3):1–22. doi: 10.1007/s12560-024-09594-3 (PMC11422458; doi:10.1007/s12560-024-09594-3)
Supplement: Supplementary file 3 — Supplementary file3 (DOCX 33 KB) [file 12560_2024_9594_MOESM3_ESM.docx]

| Virus | Ref | Name | Sequence (5’🡺3’) | Final PCR conc (µM) | Thermal profile | Gene  target | Amplicon  size (bp) | Slope of the standard curve | PCR efficiency (%) | R^2^ of the standard curve |
| --- | --- | --- | --- | --- | --- | --- | --- | --- | --- | --- |
| HAdV | **1, 2** | AdF | Fwd:CWTACATGCACATCKCSGG | 0.9 | 10min at 95°C, 40 cycles (15s at 95°C and 1min at 60°C) | hexon | 69 | -3.476 | 0.9394 | 0.9934 |
|  |  | AdR | Rev:CRCGGGCRAAYTGCACCAG | 0.9 |  |  |  |  |  |  |
|  |  | AdP1 | Prob: 6-FAM-CCGGGCTCAGGTACTCCGAGGCGTCCT-BHQ1 | 0.225 |  |  |  |  |  |  |
| JCPyV | **3** | JE3F | Fwd:ATGTTTGCCAGTGATGATGAAAA | 0.4 | 10min at 95°C, 40 cycles (15s at 95°C and 1min at 60°C) | large t-antigen | 97 | -3.272 | 1.0121 | 0.9935 |
|  |  | JE3R | Rev: GGAAAGTCTTTAGGGTCTTCTACCTTT | 0.4 |  |  |  |  |  |  |
|  |  | JE3P | Prob: 6-FAM-AGGATCCCAACACTCTACCCCACCTAAAAAGA-BHQ1 | 0.12 |  |  |  |  |  |  |
| Sendai virus (SeV) | **4** | Sendai_F | Fwd: CAGAGGAGCACAGTCTCAGTGTTC | 0.3 | RT at 48°C for 60 min and 95°C for 10 min followed by 45 cycles of 95°C for 15 sec and 60°C for 1 min | Sendai virus nucleocapsid (NP) | 123 | -3.549 | 0.9132 | 0.9905 |
|  |  | Sendai_R | Rev: TCTCTGAGAGTGCTGCTTATCTGTGT | 0.3 |  |  |  |  |  |  |
|  |  | Sendai_P | Prob: 6-FAM-TGCATCATCAGTCACACTTGGGCCTAGTA-BHQ | 0.1 |  |  |  |  |  |  |
| HAstV MLB1 | **5** | MLB1_ORF2 | Fwd: GGTCTTGGAGCYCGAATTC | 0.6 | RT at 55 °C for 60 min, 95 °C for 15 min, 45 cycles of 15s at 95°C and 1 min at 55 °C. | Capsid protein | 68 | -3.053 | 1.126 | 0.9875 |
|  |  | MLB1_ORF2_R | Rev: CGCTGTTTAATGCGCCAAA | 0.6 |  |  |  |  |  |  |
|  |  | MLB1_ORF2_P | Prob: 6-FAM - TAGRGTTGGTTCAAATCT – MGBNFQ | 0.25 |  |  |  |  |  |  |
| MNV | **6,7** | G54763F | Fwd: TGATCGTGCCAGCATCGA | 0.5 | RT at 55ºC for 1 h and 95ºC for 5 min, followed by 45 cycles at 95ºC for 15 s, 60ºC for 1 min, and 65ºC for 1 min | RdRp | 101 | -3.384 | 0.9747 | 0.9958 |
|  |  | G54863R | Rev: GTTGGGAGGGTCTCTGAGCAT | 0.9 |  |  |  |  |  |  |
|  |  | G54808P | Prob: 6-FAM-CTACCCACCAGAACCCCTTTGAGACTC-BHQ | 0.25 |  |  |  |  |  |  |
| MS2 | **8,9** | pecson-2F | AAGGTGCCTACAAGCGAAGT | 1 | RT at 55°C for 1h, 5min at 95°C, 40 cycles (15s at 95°C, 1min at 60°C and 1min at 65°C) | Maturation protein | 335 | -3.384 | 0.9746 | 0.9994 |
|  |  | pecson-2R | TTCGTTTAGGGCAAGGTAGC | 1 |  |  |  |  |  |  |
|  |  | PecP-2 | 6-FAM-ATCGTGGGGTCGCCCGTACG-BHQ1 | 0.25 |  |  |  |  |  |  |
| RoV | **10** | NSP3-F | ACCATCTWCACRTRACCCTCTATGAG | 0.4 | Pre-treatment of dsRNA extraction 99ºC-5 min and ice for 5 min. RT at 48ºC for 30 min, 10 min at 95ºC, 40 cycles (15s at 95ºC, 1 min at 60ºC) | Rotavirus Non structural protein 3 (Segment 7) | 95 | -3.388 | 0.9733 | 0.9962 |
|  |  | NSP3-R | GGTCACATAACGCCCCTATAGC | 0.4 |  |  |  |  |  |  |
|  |  | NSP3-P | 6-FAM-AGTTAAAAGCTAACACTGTCAAA- MGB | 0.4 |  |  |  |  |  |  |
| NoVGII | **11,12** | QNIF2d (300) | ATGTTCAGRTGGATGAGRTTCTCWGA | 0.5 | RT at 55ºC for 1h, 5 min at 95ºC, 45 cycles (15s at 95ºC, 1 min at 60ºC, 1min at 65ºC) | RdRp/VP1 | 89 | -3.441 | 0.9526 | 0.9959 |
|  |  | COG2R (301) | TCGACGCCATCTTCATTCACA | 0.9 |  |  |  |  |  |  |
|  |  | QNIFS (302) | 6-FAM-AGCACGTGGGAGGGCGATCG-TAMRA | 0.25 |  |  |  |  |  |  |
| HAV | **13** | HAV68 (303) | TCACCGCCGTTTGCCTAG | 0.5 | RT at 55ºC for 1h, 5 min at 95ºC, 45 cycles (15s at 95ºC, 1 min at 60ºC, 1min at 65ºC) | 5’UTR | 178 | -3.344 | 0.991 | 0.9969 |
|  |  | HAV240 (304) | GGAGAGCCCTGGAAGAAAG | 0.9 |  |  |  |  |  |  |
|  |  | HAV150 (305) | 6-FAM-CCTGAACCTGCAGGAATTAA-MGB | 0.25 |  |  |  |  |  |  |
| MRV | **18,19** | Reo_F_QIU | AGTTGCTGAACGCAAATTATTTTG | 0.9 | Pre-treatment of dsRNA extraction 99ºC-5 min and ice for 5 min.RT at 48 °C for 30 min, 5 min at 95ºC, cycles (15s at 95°C, 1 min at 60ºC) | M1 | 82 | -3.541 | 0.9161 | 0.9829 |
|  |  | Reo_R_QIU | TGCGAATCATCAGATTAACCTCTGT | 0.9 |  |  |  |  |  |  |
|  |  | Reo_P_QIU | 5-FAM-TATTGCGACTAAAAATACC-MGB | 0.25 |  |  |  |  |  |  |
| Phi 6 | **16** | Primer_F | TGGCGGCGGTCAAGAGC | 1 | Pre-treatment of dsRNA extraction 99ºC-5 min and ice for 5 min. RT at 55°C for 1h, 95°C for 5 min followed by 40 cycles of 95°C for 15 s and 60°C for 1 min (adapted for the kit) | P8 protein (Nucleocapsid). Segment S | 100 | -3.588 | 0.8999 | 0.9988 |
|  |  | Primer_R | GGATGATTCTCCAGAAGCTGCTG | 1 |  |  |  |  |  |  |
|  |  | Probe | 5-FAM-CGGTCGTCGCAGGTCTGACACTCGC-BHQ1 | 0.3 |  |  |  |  |  |  |
| AAV-2 | **17** | fwd ITR | Fwd:5′-GGAACCCCTAGTGATGGAGTT-3′ | 0.1 | 50°C for 2 min, followed by 95°C for 10 min, and 40 cycles (95°C for 1 min and annealing/extension at 60°C for 1 min) | Inverted terminal repeats (ITRs) | 62 | -3.399 | 0.9689 | 0.9981 |
|  |  | rev ITR | Rev: 5′-CGGCCTCAGTGAGCGA-3′ | 0.34 |  |  |  |  |  |  |
|  |  | AAV2 ITR_P | Prob: 5′-FAM-CACTCCCTCTCTGCGCGCTCG-BBQ-3 | 0.1 |  |  |  |  |  |  |
| EV | **18,19,20** | EV_UTR_F | Fwd: ACCGGATGGCCAATCCAA | 0.3 | RT at 55°C for 1h, followed by 5 min at 95ºC and 45 cycles (15s at 95°C 60s at 60ºC) | 5’UTR | 196 | -3.404 | 0.967 | 0.9908 |
|  |  | EV_UTR_R | Rev: CCTCCGGCCCCTGAATG | 0.9 |  |  |  |  |  |  |
|  |  | EV_Probe | Prob: 6-FAM-CGGAACCGACTACTTTGGGTGTCCGT-TAMRA | 0.1 |  |  |  |  |  |  |
| T4 | **21** | T4_F (317) | Fwd : ACTGGCCAGGTATTCGCA | 0.063 | 95°C for 30 seconds followed by 40 cycles (95°C for 5s, 60ºC for 20s and 72ºC for 15s) | Major capsid protein | 74 ^Ω^ | -3.216 | 1.046 | 0.9992 |
|  |  | T4_R (318) | Rev : ATGCTTCTTTAGCACCGGCA | 0.125 |  |  |  |  |  |  |

**References**

1. Hernroth, B.E., Conden-Hansson, A.-C., Rehnstam-Holm, A.-S., Girones, R., Allard, A.K., 2002. Environmental factors influencing human viral pathogens and their potential indicator organisms in the blue mussel, Mytilus edulis: the first Scandinavian report. Applied and environmental microbiology 68, 4523–33.

2. Bofill-Mas, S., Albinana-Gimenez, N., Clemente-Casares, P., Hundesa, A., Rodriguez-Manzano, J., Allard, A., Calvo, M., Girones, R., 2006. Quantification and stability of human adenoviruses and polyomavirus JCPyV in wastewater matrices. Applied and environmental microbiology 72, 7894–6. https://doi.org/10.1128/AEM.00965-06

3. Pal, A., Sirota, L., Maudru, T., Peden, K., Lewis Jr., A.M., 2006. Real-time, quantitative PCR assays for the detection of virus-specific DNA in samples with mixed populations of polyomaviruses. J Virol Methods 135, 32–42. https://doi.org/S0166-0934(06)00045-0 [pii]\r10.1016/j.jviromet.2006.01.018

4. Wagner, A.M., Loganbill, J.K., Besselsen, D.G., 2003. Detection of sendai virus and pneumonia virus of mice by use of fluorogenic nuclease reverse transcriptase polymerase chain reaction analysis. Comparative medicine 53, 173–7

5. Cordey, S., Vu, D.-L., Zanella, M.-C., Turin, L., Mamin, A., Kaiser, L., 2017. Novel and classical human astroviruses in stool and cerebrospinal fluid: comprehensive screening in a tertiary care hospital, Switzerland. Emerging microbes & infections 6, e84. https://doi.org/10.1038/emi.2017.71

6. Rawsthorne, H., Phister, T.G., Jaykus, L.-A., 2009. Development of a fluorescent in situ method for visualization of enteric viruses. Applied and environmental microbiology 75, 7822–7. https://doi.org/10.1128/AEM.01986-09.

7. Schultz, A.C., Uhrbrand, K., Nørrung, B., Dalsgaard, A., 2012. Inactivation of Norovirus Surrogates on Surfaces and Raspberries by Steam-Ultrasound Treatment. Journal of Food Protection 75, 376–381. https://doi.org/10.4315/0362-028X.JFP-11-271.

8. Pecson, B.M., Martin, L.V., Kohn, T., 2009. Quantitative PCR for determining the infectivity of bacteriophage MS2 upon inactivation by heat, UV-B radiation, and singlet oxygen: advantages and limitations of an enzymatic treatment to reduce false-positive results. Applied and environmental microbiology 75, 5544–54. https://doi.org/10.1128/AEM.00425-09

9. Carratalà, A., Rodriguez-Manzano, J., Hundesa, A., Rusiñol, M., Fresno, S., Cook, N., Girones, R., 2013. Effect of temperature and sunlight on the stability of human adenoviruses and MS2 as fecal contaminants on fresh produce surfaces. International journal of food microbiology 164, 128–34. https://doi.org/10.1016/j.ijfoodmicro.2013.04.007

10. Zeng, S.Q., Halkosalo, A., Salminen, M., Szakal, E.D., Puustinen, L., Vesikari, T., 2008. One-step quantitative RT-PCR for the detection of rotavirus in acute gastroenteritis. Journal of Virological Methods. https://doi.org/10.1016/j.jviromet.2008.08.004

11. 14. Kageyama, T., Kojima, S., Shinohara, M., Uchida, K., Fukushi, S., Hoshino, F.B., Takeda, N., Katayama, K., 2003. Broadly reactive and highly sensitive assay for Norwalk-like viruses based on real-time quantitative reverse transcription-PCR. J Clin Microbiol 41, 1548–1557. https://doi.org/10.1128/JCM.41.4.1548

12. Loisy, F., Atmar, R.L., Guillon, P., Le Cann, P., Pommepuy, M., Le Guyader, F.S., 2005. Real-time RT-PCR for norovirus screening in shellfish. Journal of Virological Methods. https://doi.org/10.1016/j.jviromet.2004.08.023

13. Costafreda, M.I., Bosch, A., Pintó, R.M., 2006. Development, evaluation, and standardization of a real-time TaqMan reverse transcription-PCR assay for quantification of hepatitis A virus in clinical and shellfish samples. Applied and Environmental Microbiology. https://doi.org/10.1128/AEM.02660-05

18. Qiu, Y., Lee, B.E., Neumann, N., Ashbolt, N., Craik, S., Maal-Bared, R., Pang, X.L., 2015. Assessment of human virus removal during municipal wastewater treatment in Edmonton, Canada. Journal of Applied Microbiology 119, 1729–1739. https://doi.org/10.1111/jam.12971

19. Qiu, Y., Li, Q., Lee, B.E., Ruecker, N.J., Neumann, N.F., Ashbolt, N.J., Pang, X., 2018. UV inactivation of human infectious viruses at two full-scale wastewater treatment plants in Canada. Water Research 147, 73–81. https://doi.org/10.1016/j.watres.2018.09.057

16. Gendron, L., Verreault, D., Veillette, M., Moineau, S., Duchaine, C., 2010. Evaluation of Filters for the Sampling and Quantification of RNA Phage Aerosols. Aerosol Science and Technology 44, 893–901. https://doi.org/10.1080/02786826.2010.501351

17. Aurnhammer, C., Haase, M., Muether, N., Hausl, M., Rauschhuber, C., Huber, I., Nitschko, H., Busch, U., Sing, A., Ehrhardt, A., Baiker, A., 2012. Universal Real-Time PCR for the Detection and Quantification of Adeno-Associated Virus Serotype 2-Derived Inverted Terminal Repeat Sequences. Human Gene Therapy Methods 23, 18–28. https://doi.org/10.1089/hgtb.2011.034

18. Tsai, Y.L., Sobsey, M.D., Sangermano, L.R., Palmer, C.J., 1993. Simple method of concentrating enteroviruses and hepatitis a virus from sewage and ocean water for rapid detection by reverse transcriptase- polymerase chain reaction. Applied and Environmental Microbiology. https://doi.org/10.1002/hep.1840160442

19. Monpoeho, S., Dehée, A., Mignotte, B., Schwartzbrod, L., Marechal, V., Nicolas, J.C., Billaudel, S., Férré, V., 2000. Quantification of enterovirus RNA in sludge samples using single tube real-time RT-PCR. BioTechniques. https://doi.org/10.2144/000113156

20. Fout, G.S., Cashdollar, J.L., Griffin, S.M., Brinkman, N.E., Varughese, E.A., Parshionikar, S.U., 2016. EPA Method 1615. Measurement of Enterovirus and Norovirus Occurrence in Water by Culture and RT-qPCR. Part III. Virus Detection by RT-qPCR. Journal of Visualized Experiments. https://doi.org/10.3791/52646

21. Kłopot, A., Zakrzewska, A., Lecion, D., Majewska, J.M., Harhala, M.A., Lahutta, K., Kaźmierczak, Z., Łaczmański, Ł., Kłak, M., Dąbrowska, K., 2017. Real-Time qPCR as a Method for Detection of Antibody-Neutralized Phage Particles. Frontiers in microbiology 8, 2170. https://doi.org/10.3389/fmicb.2017.02170
