# Supplementary material for: Comparison of Three Viral Nucleic Acid Preamplification Pipelines for Sewage Viral Metagenomics
Source: Food Environ Virol. 2024 Apr 22;16(3):1–22. doi: 10.1007/s12560-024-09594-3 (PMC11422458; doi:10.1007/s12560-024-09594-3)
Supplement: Supplementary file 4 — Supplementary file4 (PDF 115 KB) [file 12560_2024_9594_MOESM4_ESM.pdf]

ITEM TO CHECK	Provided (Y/N)	CHECKLIST
**EXPERIMENTAL DESIGN**		
Definition of experimental and control groups	Y	In material and methods
Number within each group	Y	In material and methods
**SAMPLE**		
Description	Y	In material and methods
Microdissection or macrodissection	N	N/A
Processing procedure	Y	In material and methods
If frozen - how and how quickly?	Y	In material and methods
If fixed - with what, how quickly?	N	N/A
Sample storage conditions and duration	Y	In material and methods
**NUCLEIC ACID EXTRACTION**		
Procedure and/or instrumentation	Y	In material and methods
Name of kit and details of any modifications	Y	In material and methods
Details of DNase or RNAse treatment	N	In material and methods
Contamination assessment (DNA or RNA)	Y	In material and methods
Nucleic acid quantification	N	Not performed
Instrument and method	N	Not performed
RNA integrity method/instrument	N	Not performed
RIN/RQI or Cq of 3' and 5' transcripts	N	Not performed
Inhibition testing (Cq dilutions, spike or other)	Y	In material and methods
**REVERSE TRANSCRIPTION**		
Complete reaction conditions	Y	In material and methods
Amount of RNA and reaction volume	Y	In material and methods
Priming oligonucleotide (if using GSP) and concentration	Y	In material and methods
Reverse transcriptase and concentration	Y	In material and methods
Temperature and time	Y	provided in Supplementary material
**qPCR TARGET INFORMATION**		
Sequence accession number	Y	provided in Supplementary material
Amplicon length	Y	provided in Supplementary material
In silico specificity screen (BLAST, etc)	N	not provided
Location of each primer by exon or intron (if applicable)	N	N/A
What splice variants are targeted?	N	N/A
**qPCR OLIGONUCLEOTIDES**		
Primer sequences	Y	provided in Supplementary material
Probe sequences	Y	provided in Supplementary material
Location and identity of any modifications	N	N/A
**qPCR PROTOCOL**		
Complete reaction conditions	Y	provided in Supplementary material
Reaction volume and amount of RNA	Y	provided in material and methods and Supplementary material
Primer, (probe), Mg++ and dNTP concentrations	Y	provided in SI and according to kit instructions
Polymerase identity and concentration	Y	According to kit instructions
Buffer/kit identity and manufacturer	Y	According to kit instructions
Additives (SYBR Green I, DMSO, etc.)	Y	According to kit instructions
Complete thermocycling parameters	Y	provided in SI
Manufacturer of qPCR instrument	Y	provided in materials and methods
**qPCR VALIDATION**		
Specificity (gel, sequence, melt, or digest)	N	N/A
For SYBR Green I, Cq of the NTC	N	N/A
Standard curves with slope and y-intercept	Y	provided in Supplementary material
PCR efficiency calculated from slope	Y	provided in Supplementary material
R^2 of standard curve	Y	provided in Supplementary material
Linear dynamic range	N	Not assessed
Cq variation at lower limit	N	Not determined
Evidence for limit of detection	N	N/A
**DATA ANALYSIS**		
qPCR analysis program (source, version)	Y	In material and methods
Cq method determination	Y	In material and methods
Outlier identification and disposition	N	N/A
Results of NTCs	Y	provided in results section
Justification of number and choice of reference genes	N	N/A
Description of normalisation method	N	N/A
Number and concordance of biological replicates	Y	In material and methods
Number and stage (RT or qPCR) of technical replicates	Y	In material and methods
Repeatability (intra-assay variation)	N	N/A
Statistical methods for result significance	N	N/A
Software (source, version)	N	N/A
