# Supplementary material for: Comparison of Three Viral Nucleic Acid Preamplification Pipelines for Sewage Viral Metagenomics
Source: Food Environ Virol. 2024 Apr 22;16(3):1–22. doi: 10.1007/s12560-024-09594-3 (PMC11422458; doi:10.1007/s12560-024-09594-3)
Supplement: Supplementary file 8 — Supplementary file8 (DOCX 17 KB) [file 12560_2024_9594_MOESM8_ESM.docx]

| Sample | Initial paired end reads | Removed reads after quality (%) | Used reads | Non-viral reads (%) | reads used for assembly  (%) | Mapped reads into contigs |
| --- | --- | --- | --- | --- | --- | --- |
| Spiked PBS Total | 2365916 | 60574 (2%) | 2305342 | 1763510 (76%) | 541832 (36%) | 196248 |
| Spiked PBS_DNA | 3959380 | 62938 (1%) | 3896442 | 2911016 (74%) | 985426 (21%) | 209458 |
| Spiked PBS RNA | 3039344 | 7294 (0%) | 3032050 | 440698 (14%) | 2591352 (96%) | 2511866 |
| Spiked sewage Total | 3283454 | 43854(1%) | 3239600 | 2246662 (69%) | 992938 (25%) | 256236 |
| Spiked sewage DNA | 3738750 | 44622 (1%) | 3694128 | 2616184 (70%) | 1077944 (25%) | 272274 |
| Spiked sewage RNA | 2748818 | 6128 (0%) | 2742690 | 1636564 (59%) | 1106126 (72%) | 798278 |
| Native sewage sample 1 Total | 3283454 | 43854 (1%) | 3239600 | 2246662 (69%) | 992938 (25%) | 256236 |
| Native sewage sample 1 DNA | 3738750 | 44622 (1%) | 3694128 | 2616184 (70%) | 1077944 (25%) | 272274 |
| Native sewage sample 1 RNA | 2748818 | 6128 (0%) | 2742690 | 1636564 (59%) | 1106126 (72%) | 798278 |
| Native sewage sample 2 Total | 1995410 | 35514 (1%) | 1959896 | 1336872 (68%) | 623024 (24%) | 152305 |
| Native sewage sample 2 DNA | 2010582 | 41076 (2%) | 1969506 | 1363372 (69%) | 606134 (30%) | 185962 |
| Native sewage sample 2 RNA | 2331744 | 100038 (4%) | 2231706 | 1713702 (76%) | 518004 (35%) | 183984 |
| Native sewage sample 3 Total | 2181682 | 38742 (1%) | 2142940 | 1591494 (74%) | 551430 (35%) | 874570 |
| Native sewage sample 3 DNA | 1949142 | 29338 (1%) | 1919804 | 1420062 (73%) | 499742 (36%) | 182756 |
| Native sewage sample 3 RNA | 2173920 | 74544 (3%) | 2099376 | 1533324 (73%) | 566052 (25%) | 145147 |
